# MSD1 regulates pedicellate spikelet fertility in sorghum through the jasmonic acid pathway

**DOI:** 10.1038/s41467-018-03238-4

**Published:** 2018-02-26

**Authors:** Yinping Jiao, Young Koung Lee, Nicholas Gladman, Ratan Chopra, Shawn A. Christensen, Michael Regulski, Gloria Burow, Chad Hayes, John Burke, Doreen Ware, Zhanguo Xin

**Affiliations:** 1 U.S. Department of Agriculture-Agricultural Research Service, Plant Stress and Germplasm Development Unit, Cropping Systems Research Laboratory, Lubbock, TX 79415 USA; 2 Cold Spring Harbor Laboratory, Cold Spring Harbor, NY 11724 USA; 3 Division of Biological Sciences and Institute for Basic Science, Wonkwang University, Iksan, 54538 South Korea; 4 Chemistry Research Unit, USDA-ARS, 1700 S.W. 23rd Drive, Gainesville, FL 32608 USA; 5 U.S. Department of Agriculture-Agricultural Research Service, NEA Robert W. Holley Center for Agriculture and Health, Cornell University, Ithaca, NY 14853 USA

## Abstract

Grain number per panicle (GNP) is a major determinant of grain yield in cereals. However, the mechanisms that regulate GNP remain unclear. To address this issue, we isolate a series of sorghum [*Sorghum bicolor* (L.) Moench] *multiseeded* (*msd*) mutants that can double GNP by increasing panicle size and altering floral development so that all spikelets are fertile and set grain. Through bulk segregant analysis by next-generation sequencing, we identify *MSD1* as a TCP (Teosinte branched/Cycloidea/PCF) transcription factor. Whole-genome expression profiling reveals that jasmonic acid (JA) biosynthetic enzymes are transiently activated in pedicellate spikelets. Young *msd1* panicles have 50% less JA than wild-type (WT) panicles, and application of exogenous JA can rescue the *msd1* phenotype. Our results reveal a new mechanism for increasing GNP, with the potential to boost grain yield, and provide insight into the regulation of plant inflorescence architecture and development.

## Abstract (web summary)

Inflorescence architecture affects crop grain yield. Here, the authors deploy whole-genome sequencing-based bulk segregant analysis to identify the causal gene of a sorghum *multi-seeded* (*msd*) mutant and suggest MSD1 regulating the fertility of the pedicellate spikelets through jasmonic acid pathway.

## Introduction

Sorghum [*Sorghum bicolor* (L.) Moench], is a versatile C_4_ grass with high efficiency in conversion of solar energy, superior drought tolerance relative to most other crops, and multiple uses as livestock feed, starch ethanol production, and human food. It is the fifth most important cereal crop in both acreage and production worldwide^1, 2^. In light of its compact genome (~730 Mb), which has been completely sequenced, sorghum is also an attractive functional genomics model for maize, sugarcane, *Miscanthus*, and other C_4_ bioenergy crops with complex genomes^3–6^.

**Fig. 1:**
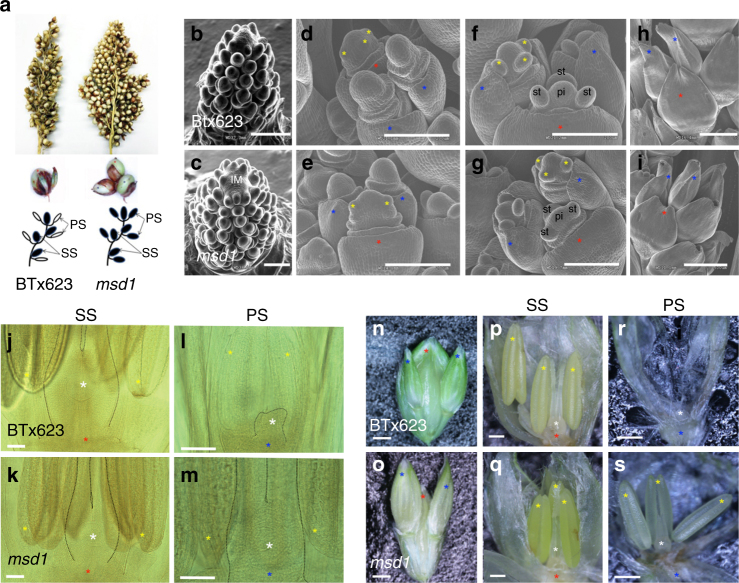
*MSD1* suppresses floral organ PS at developmental stage 4. **a** Photographs of primary branches of the panicle (adaxial side) of BTx623 (wild type, WT) and *msd1*. Images immediately below show terminal spikes: WT has one sessile grain, whereas *msd1* has three grains, one sessile and two pedicellate. The diagram provides a schematic representation of a secondary branch depicting SS and PS in WT and *msd1*: left, WT; right, *msd1*. SS sessile spikelet, PS pedicellate spikelet. **b**–**i** Detailed scanning electron microscopy analysis of inflorescence development, comparing WT and *msd1*. **b**, **c** Inflorescence meristem (IM) of WT (**b**) and *msd1* (**c**). Bar: 500 μm; (**d**,** e**) floral transition in WT (**d**) and *msd1* (**e**). Bar: 200 μm; three young stamen primordia initiate in SS of both WT and *msd1* (**d**, **e**); (**f**, **g**) developing stamen and pistil primordia in PS and SS of BTx623 and *msd1* (**g**) Bar: 200 μm. In **f** and **g**, ‘st’ indicates stamen primordia and ‘pi’ indicates pistil primordia; **h**, **i** young PS and SS at developmental stage 4. Bar: 200 μm. Yellow asterisks in panels **d**–**i** indicate young stamen primordia. **j**–**m** Photomicrographs (lateral view) of floral organ development at stage 4 in PS and SS using cleared spikelets (see Methods). Artificial contour lines were added to emphasize carpel and ovary. Ovary was fully developed only in SS of WT but in both SS and PS of *msd1*. White asterisk indicates ovary, and yellow asterisks indicate anthers. Bar: 100 μm. **n**–**s** Fully developed (mature) spike of WT (**n**) and *msd1* (**o**) at stage 5; dissected mature (lemma and palea pulled away) SS of WT (**p**) and *msd1* (**q**); and mature PS of WT (**r**) and *msd1* (**s**). White asterisk indicates ovary, and yellow asterisks indicate anthers. Bar: 1 mm. **d**–**s** Red asterisks indicate SS, and blue asterisks indicate PS

Grain number per panicle (GNP) is one of the most important contributors to grain yield in sorghum and other crops^7^, and this property is related to inflorescence architecture^1, 8^. The sorghum inflorescence exhibits a determinate panicle at the end of the shoot meristem; the main inflorescence bears primary branches at each node, and subsequent secondary or tertiary branches develop from the primary branches^9, 10^. Each inflorescence branch bears a terminal trio of floral spikelets: one sessile spikelet (SS) directly attached to the inflorescence branch and two pedicellate spikelets (PSs) attached to the inflorescence branch through a pedicel. Below the terminal spikelets, several spikelet pairs develop with one SS and one PS each. To date, in all known sorghum accessions in plant gene banks (e.g., www.ars-grin.gov) and germplasm repositories, only the SS, but not the PS, produces a perfect flower that sets grain (Fig. 1a). The PS occasionally develops anthers but no carpel tissues^11^. It remains unknown why the PS cannot develop into perfect flowers and produce viable grains.

Jasmonic acid (JA), a plant hormone derived from free α-linolenic acid with structural similarities to animal prostaglandins^12, 13^, plays diverse roles in plant adaptation to biotic and abiotic stresses and development^12, 14, 15^. During inflorescence development, JA regulates pollen development or pollen shedding in *Arabidopsis*^16^, embryo and seed development in tomato^17, 18^, spikelet formation in rice^19^, and sex determination in maize^20–22^. However, despite numerous studies focused on JA biosynthetic pathways and signaling, the regulation of JA biosynthesis during inflorescence development remains elusive.

Here we report on the characterization of a sorghum *mutliseeded1* (*msd1*) mutant that produces normal grains from both SS and PS, the latter of which is aborted in all non-mutagenized sorghum accessions. Using bulked segregant analysis of next-generation sequencing data from pooled *msd1* mutants selected from a F_2_ population, we identify the *MSD1* gene, which encodes a plant-specific transcription factor with a TCP domain. The TCP designation is coined based on the names of three founding genes: *Teosinte branched 1* (*Tb1*) in maize, *Cycloidea* (*Cyc*) in snapdragon, and *Proliferating Cell nuclear antigen binding Factor* (*PCF*) in rice^23–25^. TCP transcription factors play diverse roles in the formation of plant architecture and development^26–28^. Our results suggest that *MSD1* regulates PS development in sorghum through activation of JA biosynthesis and signaling.

## Results

### *msd1* mutants produce fertile pedicellate spikelets

In a series of *msd* mutants identified from an EMS (ethyl methanesulfonate)-induced mutant population^29^, both SS and PS developed completely fertile flowers (Fig. 1a). Furthermore, the panicles of *msd* mutants are much larger than WT due to the greater size and number of inflorescence branches^10^. Based on scanning electron microscopic analyses, sorghum panicle development can be separated into five stages (Supplementary Fig. 1). At stage 1, branching occurred from the top of the inflorescence meristem (IM) (Fig. 1b–c and Supplementary Fig. 2a). At stage 2, a triple spikelet meristem (TSM) was observed at the top of the IM, while additional branches form below the TSM (Supplementary Fig. 1, 2b). At stage 3, ovary and stamen primordia started to be observed (Fig. 1d–g and Supplementary Fig. 3a). At stage 4, SS can be clearly distinguished from PS. At stage 5, anthers and ovaries can be seen in the SS. A comparison of inflorescence development revealed no differences in panicle morphology between WT and *msd1* mutants before stage 3 (Fig. 1d–g). Floral organs initiated normally in both PS and SS in WT and *msd1* (Fig. 1b–g and supplementary Fig. 2,3). At stage 4, however, PS arrested in WT but continued to develop in *msd1* (Fig. 1h–m). At stage 5, both anthers and ovary were aborted in the PS of WT, but complete floral organs developed in both SS and PS of *msd1* (Fig. 1n–s). Together, these results indicate that floral organs initiated in both SS and PS of WT, but that PS sexual organ progression halted early on, and this suppression of floral organs was released in PS of *msd1* plants during inflorescence development.

### MSD1 encodes a TCP-domain transcription factor

**Fig. 2:**
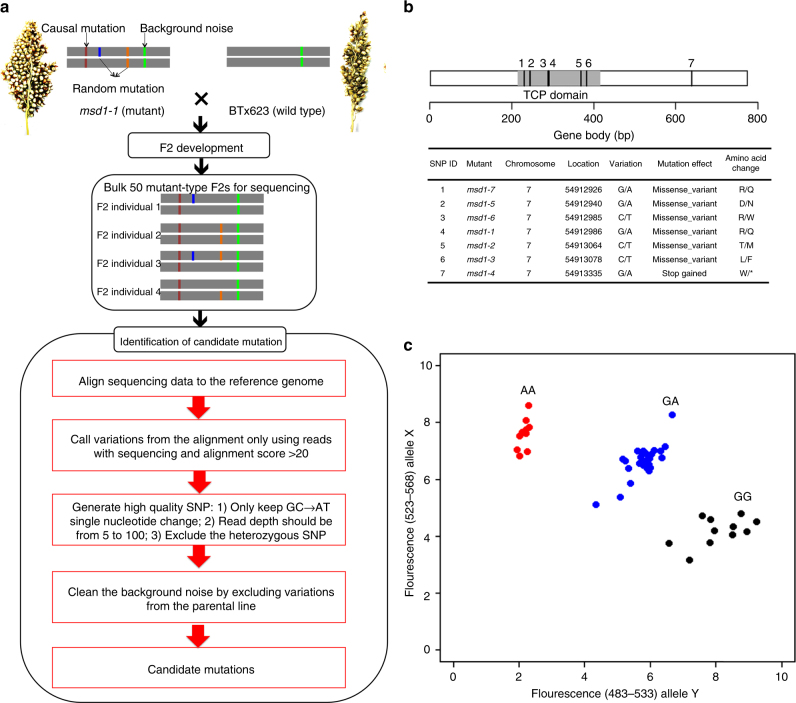
Identification of *MSD1* gene in sorghum. **a** Workflow of bulk segregant analysis combined with whole-genome sequencing, used to identify the causal gene in an *msd1-1* F_2_ population. **b** Structure of *MSD1* (Sb07g021140), depicting the CDS of 774 nucleotides and the TCP domain. The table below shows alleles of *msd1* identified by sequencing of 15 *msd* mutants. **c** Co-segregation of the *msd1-1* mutation in Sb07g021140 with the *msd* phenotype was analyzed in an F_2_ population derived from a cross of RTx437×*msd1-1*. Among the 48 individual F_2_ plants, 10 with homozygous mutations (AA) had *msd* panicles, whereas the rest (11 homozygous WT [GG] and 27 heterozygous [GA]) had WT panicles. The phenotype distribution for this population fit a 3:1 WT:*msd* ratio, with 100% co-segregation of the mutant genotype with the phenotype

To determine the genetic basis of the *msd1* phenotype, we deployed whole-genome sequencing-based bulk segregant analysis to identify the causal gene. To this end, genomic DNA from 50 homozygous *msd1-1* mutants selected from an F_2_ population were pooled and sequenced to about 27× coverage (Fig. 2a). After analysis using our in-house pipeline, we discovered 14 homozygous mutations. Whole-genome sequencing was also performed on the parental line BTx623 and an additional 17 independent homozygous *msd* mutants, including *msd1-1* and *msd1-2*: two allelic *msd1* mutants isolated from independent mutant pools. Only one gene, Sb07g021140, was homozygously mutated in the bulked F_2_ of *msd1-1* and individual *msd1-1* and *msd1-2* mutants; the *msd1-1* and *msd1-2* alleles are at different locations in this gene (Fig. 2b). Furthermore, we identified five additional unique mutations in the gene from whole-genome sequencing of the 15 other *msd* mutants (Fig. 2b). All pairwise crosses among all seven *msd1* mutants yielded the *msd1* phenotype (Supplementary Table 1).

To determine whether the trait could be expressed in other sorghum genetic backgrounds, the *msd1-1* mutant was crossed to RTx437. Among 48 individual F_2_ plants, all 10 harboring the homozygous *msd1-1* mutation (A/A) had *msd1* panicles, whereas the rest (11 homozygous WT [G/G] and 27 heterozygous [G/A]) had WT panicles, supporting the earlier conclusion that Sb07g021140 is the *MSD1* gene, and confirming that the *msd1* phenotype can be expressed in other genetic backgrounds (Fig. 2c).

*MSD1* encodes the class II TCP transcription factor *SbTCP16*, which belongs to the *CYC/TB1* subgroup of the sorghum TCP family^28^. *MSD1* is specific to monocots^28^. The mutations in the six *msd1* mutant alleles that fall within the TCP domain of Sb07g021140 (*SbTCP16*) occur at amino acid positions that are highly conserved throughout the grasses (Supplementary Fig. 4). None of the seven *msd1* mutations discovered in this study are present in diversity panels compiled by sequencing of sorghum natural populations^30, 31^.

**Fig. 3:**
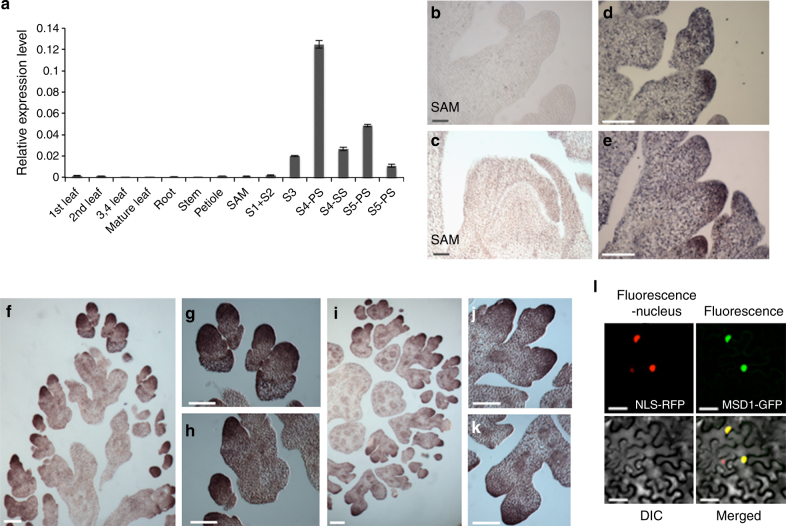
*MSD1* expression analysis in wild type. **a** Relative expression of *MSD1* from qRT-PCR in various tissue types, including leaf, root, stem, petiole, SAM, and inflorescence (stages 1+2, 3, and 4) of PS, SS (stage 4), PS (stage 5), and SS (stage 5). The bars represent mean ± SD of three biological replicates. **b**–**k** In situ hybridization of *MSD1* during inflorescence development in WT: **b** Spikelet meristem in stage 2, labeled with sense probe as a negative control. **c** Longitudinal view of shoot apical meristem. **d** Spikelet meristem near the top of meristem in stage 2, labeled with antisense probe. **e** Spikelet meristem at the bottom of meristem in stage 2. **f**–**h** Longitudinal section of floral meristem in early stage 3. **g** and **h** show magnifications of the floral meristem. **i**–**k** Transverse section of floral meristem in early stage 3. Magnifications of the floral meristem at early stage 3 in **i** are shown in **j** and **k**. **l** Cellular localization of MSD1: confocal images showing MSD1–green fluorescent protein (GFP) localization in tobacco transient assay. Top left: Nuclear Localization Sequence (NLS)-red fluorescent protein (RFP) (control); top right: MSD1-GFP; bottom left: differential interference contrast (DIC) image; bottom right: merged image of NLS-RFP and MSD1-GFP. **b**–**k** Bar: 100 μm. **I** Bar: 50 μm

Quantitative reverse transcriptase PCR (qRT-PCR) of various tissues revealed that *MSD1* expression was enriched during inflorescence development, especially at PS in stage 4 (Fig. 3a), and this expression pattern is also observed in public expression atlas data^28, 32^. To characterize the temporal and spatial expression pattern of *MSD1*, we performed RNA in situ hybridization. In a negative control hybridization using a sense probe, *MSD1* expression was undetectable in shoot apical meristem and branch primordia (Fig. 3b,c). When using an antisense probe, expression signal was observed during stage 2 in WT plants, localized to the tip of the spikelet meristem (Fig. 3d,e). Expression was maintained at the tip of the floral meristem and then expanded throughout the floral meristem including the glume at stage 3 (Fig. 3f–h). Both transverse and longitudinal sections revealed that *MSD1* expression was specifically maintained in a specific dome-like domain of the floral meristem (Fig. 3f–k). At stage 4 (Supplementary Fig. 5), although weak expression of *MSD1* was detected in the ovary of both PS and SS, a much stronger signal was observed in anthers. Consistent with the role of MSD1 as a transcription factor, an MSD1–green fluorescent protein (GFP) fusion protein exclusively localized in the nucleus in a transient assay using tobacco leaf cells (Fig. 3i), in agreement with a previous prediction^28^.

### MSD1 regulates the JA pathway

To interrogate the regulatory targets of MSD1, we performed transcriptome profiling by RNA-seq on developing panicles from four developmental stages of WT and *msd1-1* with three biological replicates (Supplementary Table 2). After discarding two replicates (*R*^2^ < 0.9 relative to another replicate), the average Pearson correlation coefficient of the three biological replicates of each developmental stage was 0.97, indicating that the remaining samples were of high quality (Supplementary Fig. 6a). The *MSD1* genotypes were also validated in the transcriptome profiling of both WT and mutant (Supplementary Fig. 6b). Among the 26,654 expressed genes in WT, 11,528 exhibited dynamic changes during panicle development. In WT, more genes were differentially expressed between SS and PS in stage 4 than in stage 5 (1848 versus 97). The number of the genes most differentially expressed between WT and *msd1* was also at stage 4 (Supplementary Table 3). The upregulated genes in *msd1* vs. WT at stage 4 in PS (Supplementary Table 4,5) were mainly involved in grain development, indicating that the floral development was set in stage 4.

**Fig. 4:**
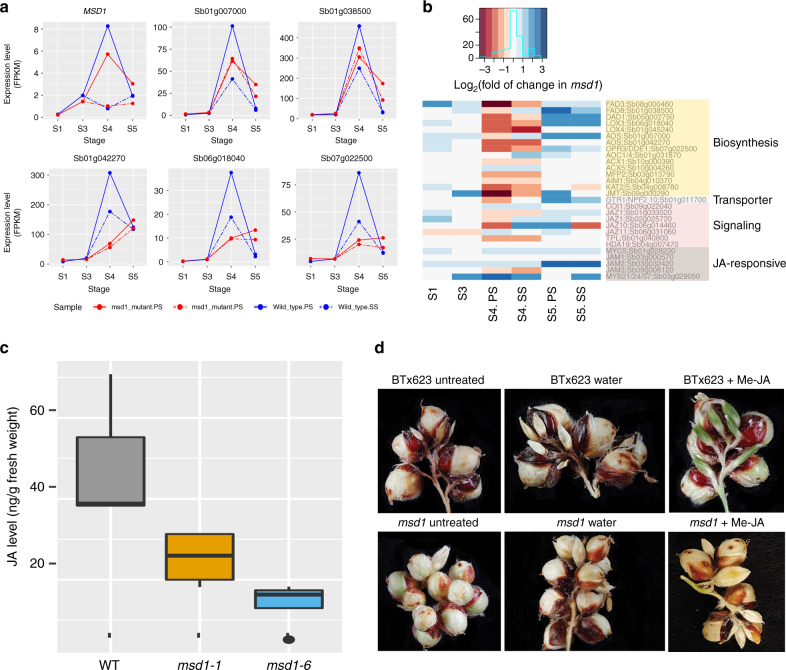
MSD1 regulates the JA pathway and exogenous Me-JA rescues the *msd1* phenotype. **a** Expression pattern of *MSD1* and five putative target genes identified in the RNA-seq analysis that are involved in jasmonic acid (JA) biosynthesis (as determined by orthologs of *Arabidopsis thaliana* JA pathway). The blue lines depict pattern of expression in WT, whereas red lines represent expression in the *msd1-1* mutant. At stages 4 and 5, solid and dotted lines indicate PS and SS, respectively. The expression level of *MSD1* clearly differed between SS and PS in stage 4 in WT. *MSD1* was differentially expressed between WT and *msd1* only at stage 4 in PS. For the five putative target genes, the two red lines almost overlap with each other, indicating that in the *msd1* mutant, the expression levels of these three genes were similar in PS and SS. **b** Heatmap of fold change in expression of sorghum orthologs of the *Arabidopsis* JA pathway genes in *msd1* mutants. Genes involved in biosynthesis were downregulated at stage 4 in PS of *msd1*. **c** Box plot showing JA concentrations from five young panicle samples from each of two *msd1* mutants and WT (BTx623). Lines from bottom to top represent minimum, first quartile, median, third quartile, and maximum; dots indicate outliers. JA levels were lower in the two mutants than in WT. **d** Photograph of adaxial side of a secondary branch of inflorescence during exogenous JA treatment of WT and *msd1-1* plants. The *msd1-1* mutant reverted to the WT phenotype following treatment with methyl jasmonate (Me-JA)

Based on the expression pattern of *MSD1* (Fig. 4a) and a study of the CYC pathway in *Antirrhinum*^25^, we established predicted expression patterns for putative regulatory targets of MSD1. Because *MSD1* expression peaked at stage 4 in PS, MSD1 may act as a suppressor that inhibits the development of this organ. Therefore, we reasoned as follows: (1) Because the target genes are mainly required at stage 4 to determine the fate of PS, their expression should peak at S4 in PS in WT plants; (2) because MSD1 suppresses the development of PS in WT but not in *msd1*, the target genes should be expressed at higher levels in PS than in SS in WT, but their expression levels in PS should be greatly reduced in *msd1*; and (3) because both PS and SS developed into grains in the mutant, the target genes should be expressed at similar levels in PS and SS during stage 4 in *msd1*. A total of 167 genes with this pattern were identified as candidate regulatory targets. Based on Gene Ontology (GO) term analysis, genes involved in JA biosynthesis and metabolism were significantly enriched in this set (Table 1). Five putative regulatory targets involved in JA biosynthesis exhibited a pattern consistent with MSD1 targets (Fig. 4a). Among the other orthologs of the *Arabidopsis* JA pathway, only genes involved in biosynthesis were downregulated at stage 4 in PS of *msd1* (Fig. 4b).

**Table 1:** The 20 most enriched biological process GO terms among putative target genes

GO term	Description	No. in candidate targets	No. in background	*P*-value^a^	FDR^a^
GO:0031408	Oxylipin biosynthetic process	9	66	6.60E-10	2.60E-07
GO:0031407	Oxylipin metabolic process	9	66	6.60E-10	2.60E-07
GO:0006633	Fatty acid biosynthetic process	12	255	7.40E-08	1.90E-05
GO:0009694	Jasmonic acid metabolic process	7	61	1.60E-07	2.60E-05
GO:0009695	Jasmonic acid biosynthetic process	7	61	1.60E-07	2.60E-05
GO:0015979	Photosynthesis	11	250	5.00E-07	6.50E-05
GO:0006629	Lipid metabolic process	25	1351	6.00E-07	6.70E-05
GO:0006631	Fatty acid metabolic process	13	387	8.60E-07	8.40E-05
GO:0019684	Photosynthesis, light reaction	8	144	3.70E-06	0.00032
GO:0009765	Photosynthesis, light harvesting	5	45	1.20E-05	0.00093
GO:0016053	Organic acid biosynthetic process	13	637	0.00014	0.0094
GO:0046394	Carboxylic acid biosynthetic process	13	637	0.00014	0.0094
GO:0009617	Response to bacterium	10	405	0.0002	0.011
GO:0032787	Monocarboxylic acid metabolic process	13	661	0.00021	0.011
GO:0009753	Response to jasmonic acid stimulus	9	362	0.0004	0.021
GO:0009698	Phenylpropanoid metabolic process	10	454	0.00048	0.023
GO:0042742	Defense response to bacterium	7	261	0.0011	0.05
GO:0008610	Lipid biosynthetic process	12	694	0.0011	0.05
GO:0009628	Response to abiotic stimulus	27	2423	0.0013	0.052
GO:0055114	Oxidation reduction	8	352	0.0015	0.057

^a^The enrichment was calculated by Hypergeometric test. Multi-test adjustment was performed by Yekutieli (FDR under dependency) method

To determine whether JA levels were regulated by MSD1, we measured the changes in JA levels in developing panicles of WT (BTx623), *msd1-1*, and *msd1-6*. In stage 3 panicles, JA levels were reduced by >50% relative to WT in both mutants (Fig. 4c). Furthermore, the *msd* phenotype could be rescued by exogenous application of 1 mM methyl-JA to the whorl of *msd1* mutants starting at stage 3 or earlier (Fig. 4d). However, no rescue occurred if methyl-JA was applied after stage 4. We also noted that prolonged methyl-JA treatments at concentrations >1 mM decreased overall panicle size and branching in comparison with controls.

## Discussion

Spikelets are specialized units of the diverse inflorescence (flower) architectures of grasses^8^. Spikelets are classified into two types based on their mode of attachment to the inflorescence branch: SSs are directly attached, while PSs are attached through a short petiole called the pedicel. SS are also referred to as ‘central’, and PS as ‘lateral’, in some grass species^33, 34^. In general, only SS can produce fertile flowers and grains, whereas PS develop either staminate floral organs or no floral organ at all^8, 10, 35^. In this study, our characterization of the sorghum *msd1* mutants, which bear fertile PS, reveals that suppression of floral organ development is regulated by the TCP transcription factor MSD1 via activation of JA biosynthesis. As described below, this suppression may be mediated through JA-induced programmed cell death.

The TCP-domain proteins are a family of plant-specific transcription factors that shape plant form and architecture^26^. MSD1 belongs to the CYC/TB1 sub-group of class II TCP-domain proteins. The founding gene of this sub-group, *Cyc*, was originally isolated from *Antirrhinum* as a regulator of flower asymmetry^25^. In WT *Antirrhinum*, *Cyc* is expressed at the dorsal (adaxial) side of the apical meristem during sepal primordia initiation and inhibits formation of the sepal, petal, and stamen. The WT flower has five sepals, five petals, four stamens, and one aborted stamen (called the staminode) on the dorsal side. By contrast, in *cyc* mutants, suppression of initiation and growth of flower organs is abolished, and consequently the staminode on the dorsal side becomes a fully developed stamen. In addition, a new stamen on the dorsal side initiates and fully develops. Thus, the *cyc* flower has six sepals, six petals, and six stamens. *Cyc* is also expressed on the dorsal side of the *Antirrhinum* flower during the late stage of development, altering its symmetry. Another well-studied member of the TCP/TB1 sub-group is the maize *Tb1* gene. Similar to CYC in *Antirrhinum*, which suppresses the initiation and development of flower organs on the dorsal side, TB1 controls apical dominance by inhibiting development of basal tillers^23^. Its rice ortholog (Os03G0706500) also functions as a suppressor of tiller development^36^. The sorghum ortholog of maize *Tb1* is Sb01g010690 (*SbTCP2*) and is induced by high planting density and far-red light enrichment, which reduces tiller development^37^. Therefore, it is likely that *SbTCP2* also mediates tiller development. However, phylogenetic analysis indicates that *MSD1* is not the ortholog of *Tb1*, and no functional study of its orthologs has been conducted in other grass species (Supplementary Fig. 7). Here we showed that, in sorghum, *MSD1* regulates fertility of PS rather than vegetative tiller.

The inflorescence of barley has a very different architecture than that of sorghum: it has a single indeterminate main inflorescence axis that produces three single-flowered spikelets in a distichous manner at each rachis internode^33^. In the ancestral two-row barley, each central fertile spikelet is accompanied by two lateral aborted spikelets, whereas in six-row barley, the two lateral spikelets are fertile. To date, five genes have been identified that contribute to the conversion of two-row to six-row barley^33, 34, 38–40^. One of them, *VRS5/INT-C*, is the barley homolog of maize *Tb1* and modifies lateral spikelet development in certain *vrs1* allele backgrounds^39^. Both INT-C and MSD1 belong to the CYC/TB1 subgroup of class II TCP domain proteins, although INT-C is more closely related to the sorghum TB1 ortholog Sb01g010690 (SbTCP2) than to MSD1 (SbTCP16). Our whole-genome sequencing of 17 independent *msd* mutants revealed no large-effect mutations in the sorghum homologs of the other barley *VRS* genes, indicating the pathways that control row number in barley and inflorescence branching and fertility of PSs in sorghum may recruit specific components in each species^39^.

It remains unknown how TCP transcription factors regulate diverse features of plant architecture. Schommer et al. (2008) showed that a group of TCP transcription factors regulate early-stage leaf morphogenesis and leaf senescence through activation of JA biosynthesis enzymes in *Arabidopsis*^41^. Similarly, our results demonstrate that MSD1 also arrests the development of PS through activation of JA biosynthesis enzymes (Fig. 4). Based on our results, we devised a regulatory model of how MSD1 represses the development of PS in WT panicles. According to this model, a development signal during stage 3 may activate the expression of *MSD1* in PS, leading to an increase in JA production. At stage 4, elevated JA level triggers a programmed cell death that leads to the arrest of further floral organ development, and eventually abortion of these tissues, in PS^42, 43^; this idea is supported by our transcriptome profiling (Supplementary Fig. 8). Consistent with the arrest of PS in WT, the top 10 most upregulated genes in the PS of *msd1-1* vs WT PS at stage 4 are members of the late embryogenesis abundant protein^44, 45^ and cupin families^46^, indicating that embryo development in WT PS essentially ceases (Supplementary Table 4 and Supplementary Fig. 8). This hypothesis is also consistent with the sex-determination function of *tassel seed 1* (*Ts1*) on the fate of tassel spikelet in maize^20^; *Ts1* encodes a plastid-targeted 13-lipooxygenase that catalyzes the first committed step in JA biosynthesis. Maize tassel spikelets are staminate because the pistil primordia abort due to TS1-mediated programmed cell death. Recent work showed that *vrs2*, a six-row barley mutant, exhibits a phenotype analogous to those of *msd* mutants in sorghum that may be regulated by the hormone balance of auxin, GA, and cytokinin^38^. Because the JA level was not monitored in the barley studies, it remains to be determined whether sorghum and barley regulate the suppression of spikelet development through the same or different mechanisms.

In summary, we revealed a mechanism by which GNP is dramatically increased by de-repression of PS development in sorghum panicles. Specifically, we showed that mutation in the TCP transcription factor MSD1 blocks JA signaling, which suppresses organ formation in PS and its development into a perfect flower, possibly by inducing programmed cell death. Reduction of the JA level in developing panicles may also increase branch number and size during panicle development, thereby greatly increasing the overall panicle size under non-limiting environmental conditions^19^. Given the widespread presence of TCP transcription factors in crop plants, these findings suggest a novel approach for increasing grain number in cereal crops.

## Methods

### Imaging of inflorescence structures in WT and mutant

Inflorescence tissues at five stages (from meristem to immature spikelets) were collected from greenhouse-grown plants and processed for scanning electron microscopy^47^. To visualize the floral organs in SS and PS, SS and PS were collected from BTx623 and the *msd1-1* mutant at stage 4 and cleared by fixing in FAA (50% ethanol, 1% formaldehyde, and 0.5% acetic acid) overnight at 4 °C. Fixed PS and SS samples were washed with an ethanol series (50, 70, 85, and 100% for at least 30 min each) and then dehydrated in 100% ethanol for 1 h. Fixed tissues were immersed in 1:1 methyl salicylate:ethanol solution for 1 h, transferred to 100% methyl salicylate solution, and soaked for an additional 1 h. For Nomarski microscopy, slides were sealed with Vaseline to keep the samples immersed in methyl salicylate solution. PS and SS organs were observed under Nomarski optics on a Leica DMRB microscope equipped with a QImaging Micropublisher 5.0 RTV.

### Identification of the causal gene by bulk sequencing

The *msd1-1* mutant was backcrossed to WT (BTx623) in a greenhouse. The WT, 17 independent *msd* mutants, and backcrossed F_2_ seeds from the *msd1-1* backcross were planted in the USDA-ARS field in Lubbock, TX. During the late grain-filling stage, 50 plants with the mutant phenotype were selected from the backcrossed F_2_ plants. Leaf samples were collected and used to prepare genomic DNA with a CTAB method followed by purification with MagAttract beads^48^. Genomic DNA was pooled in equal amounts from the 50 individual F_2_ mutants and diluted to 100 ng/µl. Pooled F_2_ genomic DNA, as well as genomic DNA from BTx623 and 17 independent homozygous *msd* mutants, was sequenced on an Illumina HiSeq2000.

Low-quality reads, adaptor sequences, and contamination were excluded from the raw reads. The remaining clean reads were aligned to the sorghum reference genome v1.4 using Bowtie2^49^. Single-nucleotide polymorphism (SNP) calling was performed with Samtools and Bcftools, using only reads with mapping and sequencing quality >20^50^. The read depth for true SNPs was set from 3 to 50 for the parental line BTx623 and individual *msd* mutants and from 5 to 100 for the *msd1-1* F_2_ population. SNPs from the F_2_ population and 17 mutants were analyzed using Ensembl Variant Effect Predictor^51^. Because EMS induces only G/C→A/T transition mutations^52^, only homozygous G/C→A/T were included in this analysis. SNPs from BTx623 were treated as background noise and filtered from all mutants and the F_2_ population.

To confirm mutations in the candidate genes, primers were designed using Primer3 (www.frodo.wi.mit.edu/primer3/input.htm). Genomic DNA from WT and each of the *msd* mutants was amplified using Phusion high-fidelity DNA polymerase (New England Biolabs, Ipswich, MA, USA). All PCR products were purified using the QIAquick PCR Purification Kit (Qiagen, Valencia, CA, USA) before use as templates for sequencing. Subsequently, nested sequencing primers that encompassed the predicted SNPs were designed and used for fluorescent Sanger sequencing on an ABI Genetic Analyzer 3130 XL (Life Technologies, Grand Island, NY, USA). To verify mutations, DNA sequences were assembled and analyzed using the DNAman software (Lynnon, San Ramon, CA, USA) at the chromatogram level.

To further confirm the causal SNPs identified by this approach, candidate genes were genotyped in 48 F_2_ individuals from the RTx437×*msd1-1* cross using a rapid genotyping method, Kompetitive Allele Specific PCR (KASP) by Design (KBioscience/LGC Genomics [www.lgcgenomics.com]), using the manufacturer’s protocols with some modifications. Briefly, the touchdown PCR for the *msd1* primers was from 65 to 57 °C, and the actual PCR was performed for 30 cycles at an annealing temperature of 57 °C. Because the *msd* phenotype was scored as a binary trait, the SNP–trait relationship was analyzed using Fisher’s exact test.

### In situ hybridization

RNA in situ hybridization was conducted according to a published protocol with slight modifications^53, 54^. Inflorescence meristem and shoot apical meristem were dissected under a microscope and fixed under vacuum (400 mm Hg) for 20 min in 4% paraformaldehyde containing 20% Tween-20. Shoot apical meristem was collected 3 weeks after germination, and IM was collected at various developmental times based on size and morphology. The ~500-bp *MSD1* transcript was amplified from BTx623 cDNA and ligated into StrataClone PCR Cloning Kit vector pSC-A-amp/kan (Agilent Technologies, Santa Clara, CA, USA). T7 and T3 RNA polymerases were used for *MSD1* in vitro transcription and hybridization. *MSD1* transcripts were detected using ~500-bp digoxigenin-labeled *MSD1* antisense or sense probe; the latter served as a negative control. Primers are listed in Supplementary Table 6.

### Localization of MSD1 in tobacco

The *MSD1* gene (without a stop codon) was subcloned into pdnor221 using BP Clonase and ligated into the destination vector pMDC83. pMDC83 harboring the 35S promoter and *MSD1* C-terminally fused to GFP was generated using LR Clonase. pMDC83-*MSD1* was transformed into *Agrobacterium tumefaciens* strain GV3101. The transformed *Agrobacterium* were cultured at 28 °C for 2 days in YEP liquid (5 g NaCl, 10 g Bacto peptone, 10 g yeast extract) supplemented with rifampicin (60 mg/ml), kanamycin (50 mg/ml), gentamycin (50 mg/ml), and hygromycin (40 mg/ml). The *Agrobacterium* were then precipitated, re-suspended in infiltration buffer (2-ethanesulfonic acid, pH 5.5, 10 mM MgSO_4_) containing 10 µM acetosyringone, and used to inoculate the abaxial epidermal side of 3–4-week-old *Nicotiana* *benthamiana* leaves. Two days after infiltration, leaves were observed for GFP and NLS-RFP fluorescence signal on a confocal microscope (ZEISS 710).

### Expression analysis

For real-time qRT-PCR, total RNA was extracted using TRIzol reagent (Life Technologies, Grand Island, NY, USA) from root, shoot apical meristem, leaf, petiole, stage 1 IM, stage 3 IM, PS, and SS at stages 4 and 5. RNA samples were treated with DNase according to the Ambion Turbo DNA-free Kit protocol (Ambion, Austin, TX, USA). cDNA was synthesized from 400 ng RNA using the Superscript III First Strand Synthesis Kit (Life Technologies), and an *MSD1*-specific primer was designed to amplify a 200–300 bp region of the transcript. Control primers were designed against *Sb-Eukaryotic Initiation Factor 4 α*(*Sb-EIF4α*). All real-time PCR analyses were performed on a C1000 Thermal Cycler (Bio-Rad, Hercules, CA, USA). Primers were tested on WT (BTx623) by quantitative PCR on a Bio-Rad CFX96 Real Time System to confirm primer quality, as determined by melting curve and amplification efficiency. Gene expression in each sample was measured in three technical replicates. Primer sequences used for qRT-PCR are provided in Supplementary Table 6.

### Transcriptome profiling

For RNA-seq analysis, panicle development was separated into different stages according to size, as described in the Results section. Three replicates (10 plants each) at each stage of development were used for tissue collection. Briefly, at panicle initiation stage 1, whole panicles were harvested from 10 plants, pooled together for each replicate, and processed for extraction of total RNA. In transition stage 3, the differentiated floral organs on the tips of panicles (one-third of the length) were harvested from 10 plants, pooled together for each replicate, and processed for extraction of total RNA. Sessile and pedicellate tissues could be separated at stages 4 and 5 using a dissecting microscope, and these tissues were harvested accordingly for each from 10 plants and pooled for each replicate. Tissues were immediately frozen in liquid nitrogen and stored at −80 °C prior to RNA extraction.

RNA was extracted from 48 samples using TRIzol reagent, and total RNA for each sample was further subjected to DNase treatment and purification using the RNeasy Mini Clean-up Kit (Qiagen). The quality of total RNA was examined on 1% agarose gels and using RNA Nanochips on an Agilent 2100 Bioanalyzer (Agilent Technologies). Samples with RNA integrity number^55^ ≥7.0 were used for library preparation. Total RNA (2 μg) was used for poly (A)^+^ selection using oligo (dT) magnetic beads (Invitrogen 610-02), eluted in 11 µl of water, and used for RNA-seq library construction with the ScriptSeq^™^ v2 kit (Epicentre SSV21124). Final libraries were amplified by 13 PCR cycles. RNA-seq of three biological replicates was carried out in the sequencing center of Cold Spring Harbor Laboratory.

RNA-seq data from each sample was first aligned to the sorghum version 1.4 reference genome using STAR^56^. Quantification of gene expression levels in each biological replicate was performed using Cufflinks^57^. The correlation coefficient among the three biological replicates for each sample was evaluated by the Pearson test in the R statistical environment. After removal of two bad samples, the biological replicates were merged together for differential expression analysis using Cuffdiff^57^. Only genes with at least five reads supported in at least sample were subjected to differential expression analysis. The cutoff for differential expression was adjusted False Discovery Rate *p* < 0.05. GO term analysis was performed with agriGO^58^ Singular Enrichment Analysis using the hypergeometric statistical test method, with significance level set to 0.01.

### JA quantification

Five samples of young panicle (0.2–0.9 cm) were collected from WT BTx623 and two *msd1* mutants for JA quantification. Samples were solvent-extracted, methylated, collected on a polymeric adsorbent using vapor-phase extraction, and analyzed using gas chromatography/isobutane chemical ion mass spectrometry^59^. For metabolite quantification, d5-JA (Sigma-Aldrich, St. Louis, MO, USA) was used as an internal standard. The JA level in each sample was normalized to the weight of the panicle and is expressed as 'ng/g fresh weight'.

### Phenotypic rescue of *msd1* with exogenous methyl-jasmonate

BTx623 or *msd1* mutant seeds were germinated and grown in 2-gallon pots with four seedlings/pot for 16-h day cycles at 24 °C in a greenhouse. Beginning at leaf collar stage 7, 1 mL of either 0.05% Tween-20 (polyethylene glycol sorbitan monolaurate) in water (control) or 0.5 mM, 1.0 mM, or 5 mM methyl-jasmonate in 0.05% Tween-20 was aspirated directly down the floral whorl using a pipette. This treatment was repeated every 48 h until the majority of control plants reached the flag leaf stage. At this point, all experimental treatments were halted . All plants were allowed to mature to at least the soft dough stage prior to evaluation of whether successful rescue had occurred. Panicles were analyzed from the soft dough through black-layer stages to account for any delay in development or seed-filling that might have occurred as a result of methyl-JA treatment. Based on preliminary results, methyl-JA concentrations of 1 mM were optimal; higher concentrations severely retarded growth and development of the plant and resulted in significantly smaller panicles than in plants subjected to lower concentrations of methyl-JA.

### Data availability

The BSA sequencing and RNA-seq data have been deposited to NCBI SRA under accession number SRP127741.

## Electronic supplementary material

Supplementary Information

Peer Review File
