# Supplementary material for: MSD1 regulates pedicellate spikelet fertility in sorghum through the jasmonic acid pathway
Source: Nat Commun. 2018 Feb 26;9:822. doi: 10.1038/s41467-018-03238-4 (PMC5826930; doi:10.1038/s41467-018-03238-4)
Supplement: Supplementary file 1 — Supplementary Information [file 41467_2018_3238_MOESM1_ESM.pdf]

Supplementary Information

Jiao and Lee *et al.*,

MSD1 Regulates Pedicellate Spikelet Fertility in Sorghum through the Jasmonic Acid Pathway

BTx623	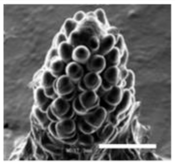	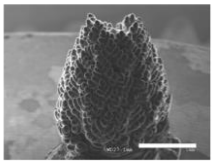	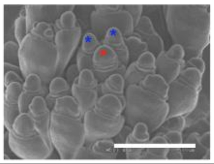	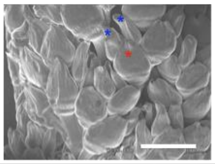	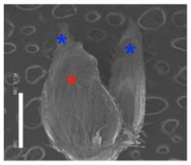
Developmental stage	Stage 1<br><br>Inflorescence meristem 1	Stage 2<br><br>Inflorescence meristem 2	Stage 3<br><br>Floral transition	Stage 4<br><br>Floral Stage 1	Stage 5<br><br>Floral Stage 2
				SS<br>Less than ~2mm<br><br>PS<br>Less than ~1mm	SS<br>>4mm<br><br>PS<br>>2mm
Length of inflorescence	1-3mm	3-5mm	5-15mm	4.5-7cm	10-15cm
Leaf number	7.3-8.3	8.3-9.3	9.3-10.3	10.3-12	13-14

Supplementary Fig. 1. Detailed description of the inflorescence developmental stages defined in this study. Top: SEM showing inflorescence developmental stages in BTx623. Bar for Stage 1 and 3: 500um; Bar for Stage 2, 4 and 5: 1mm. Stage 3-5: Red asterisks indicate sessile spikelet (SS), and blue asterisks indicate pedicellate spikelet(PS).

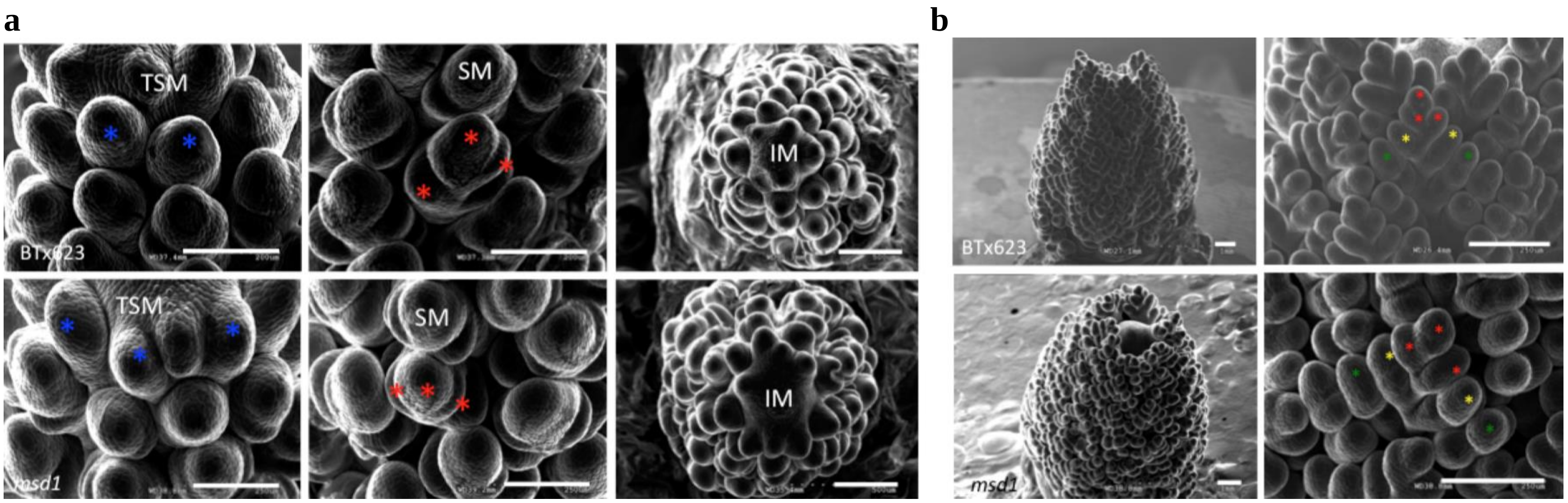

Supplementary Fig. 2. SEM comparison of the development of inflorescence meristem at stage 1 and stage 2 in wild type and *msd1* mutant. **a)** Stage 1 of WT (top panel) and *msd1* (bottom panel): left, top of inflorescence meristem; middle, bottom view of inflorescence meristem; right, overview from top. Blue asterisks indicate triple spikelet meristem (TSM), and red asterisks indicate spikelet meristem (SM). IM: inflorescence meristem. A single spikelet is observed at the top of the inflorescence meristem, and three spikelet meristems are located at the bottom of the inflorescence meristem. Bar: 200um. **b)** Stage 2 of WT BTx623 (top panel) and *msd1* (bottom panel): left, general view of inflorescence meristem; right, magnification of inflorescence meristem at stage 2. Three red asterisks indicate three spikelet meristems. Two more spikelet meristems (yellow asterisks) are observed below the three spikelet meristems (red asterisks) in both WT and *msd1*. Two additional spikelet meristems (green asterisks) are located below the two spikelet meristems (yellow asterisks) in both WT and *msd1*. Bar: 250um.

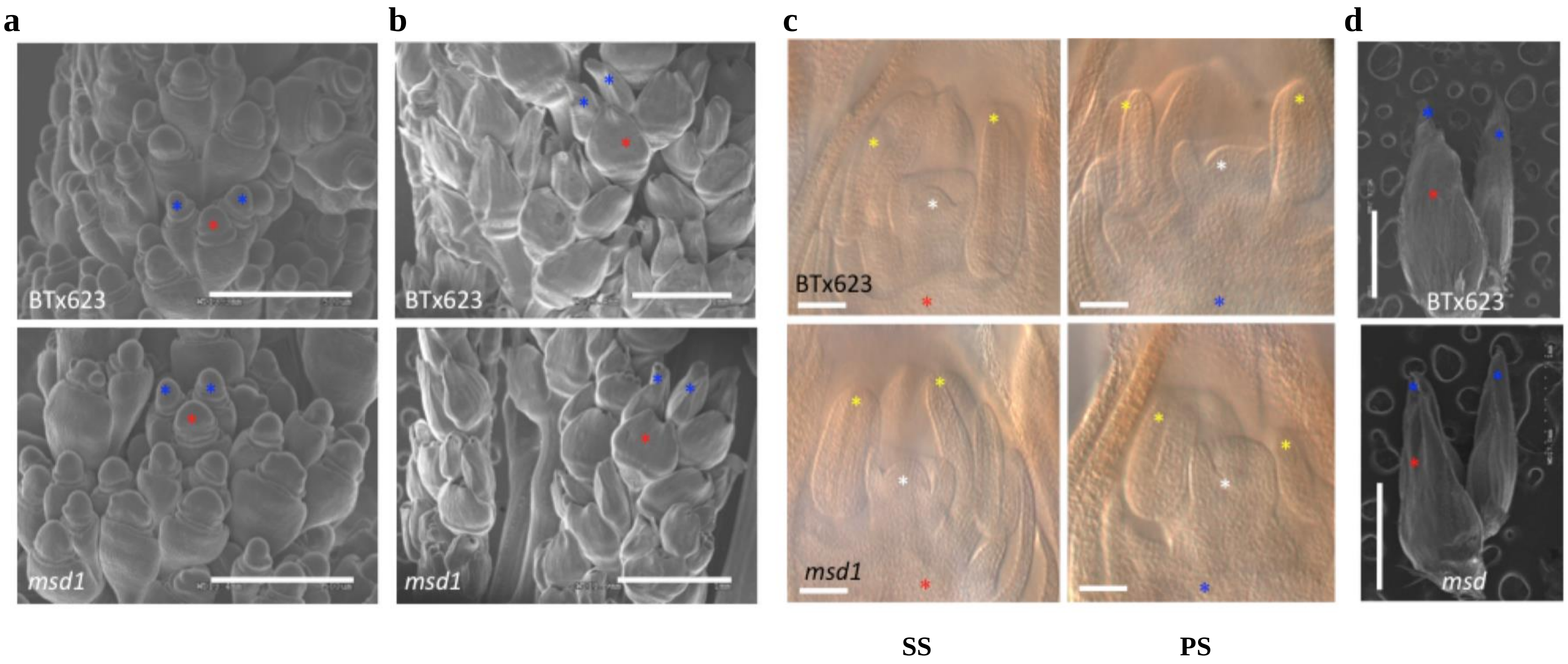

Supplementary Fig 3. Comparison of WT and *msd1* from floral transition to floret developmental stages. a) Panicle phenotype at the floral transition stage in WT (top) and *msd1* (bottom). The sessile spikelet (SS) is more expanded than the pedicellate spikelet(PS), reflecting the fact that the SS develops slightly earlier. Bar: 500um. **b)** Panicle phenotype at the stage 4 in wild type (top) and *msd1* (bottom), determined by SEM. Bar: 1mm. **c)** Panicle phenotype at stage 4 in wild type (top) and *msd1* (bottom) without dissection. Phenotype was observed under a Nomarski microscope. Top, wild type; bottom, *msd1*. Bar: 100 μm. White asterisk indicate ovary. Yellow asterisk indicate anther in BTx623 and *msd1*. **d)** Panicle phenotype at stage 5 in WT (top) and *msd1* (bottom), determined by SEM. Red asterisk indicates sessile spikelet (SS), and blue asterisk indicates pedicellate spikelet (PS). Bar: 1mm.

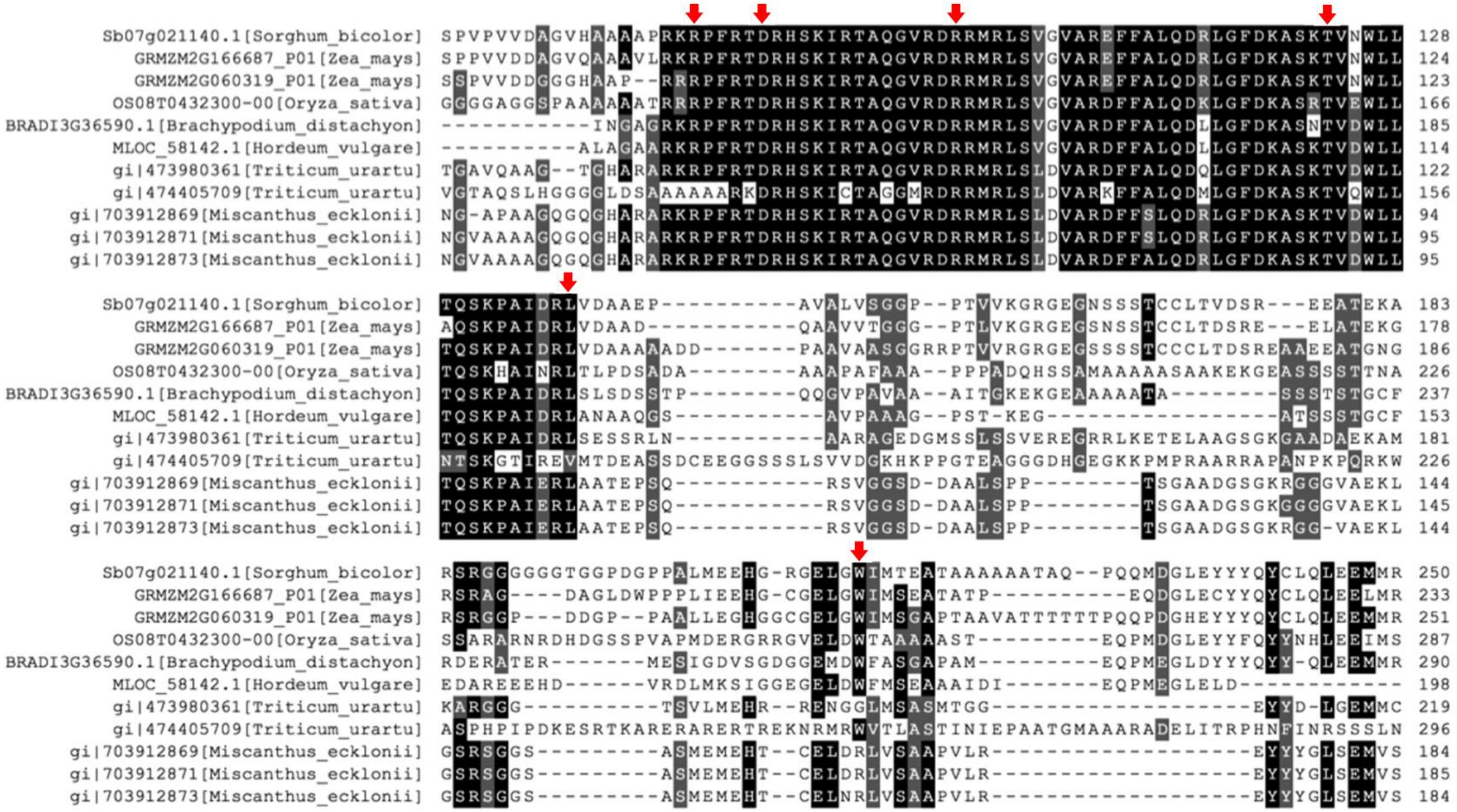

Supplementary Fig. 4. Amino acid changes caused by mutations in *msd1* mutants are conserved across the grass family. The changes are pointed by red arrows.

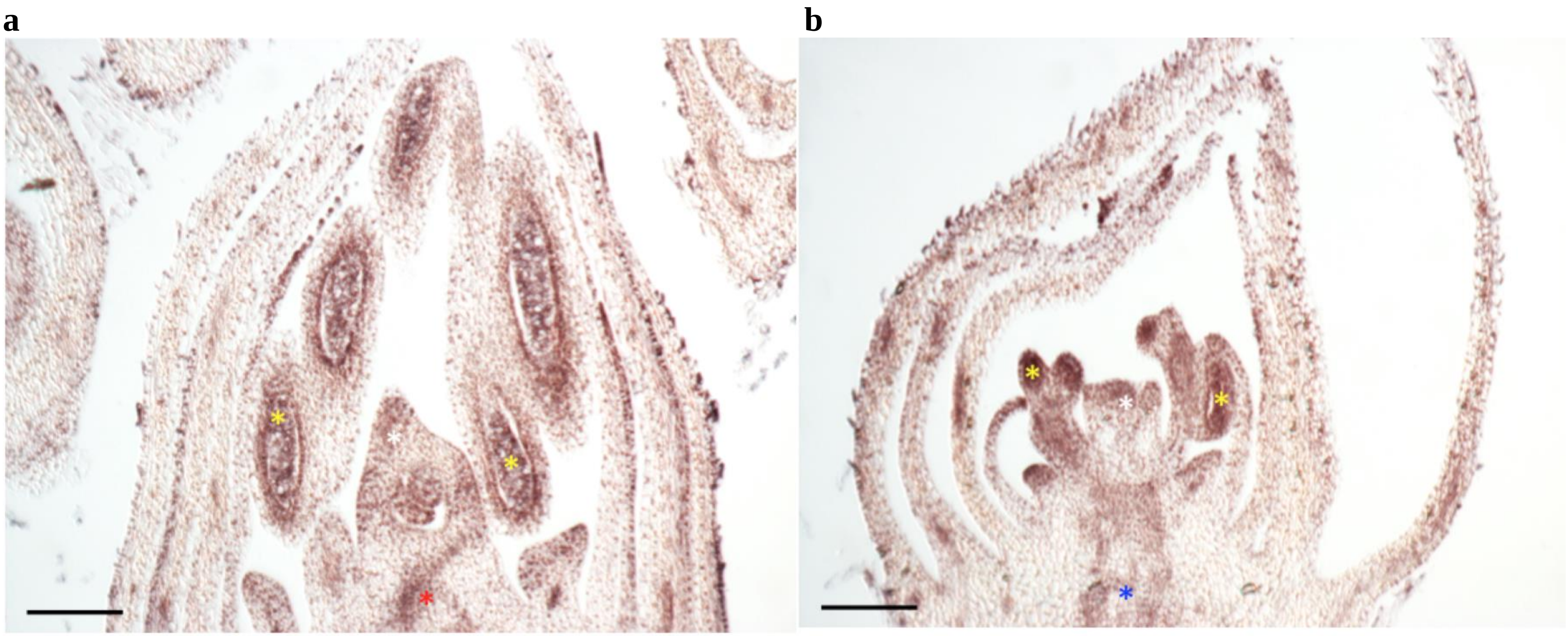

Supplementary Fig. 5. ***In situ*** **hybridization of** ***MSD1*** **at the stage 4 in WT. a)** longitudinal section of sessile spikelet (SS) at stage 4, labeled with antisense probe. **b)** Longitudinal section of pedicellate spikelet (PS) in stage 4. White asterisk indicates ovary, and yellow asterisks indicate anthers. Red asterisk indicates SS, and blue asterisk indicates PS. Bar: 100 µm.

a

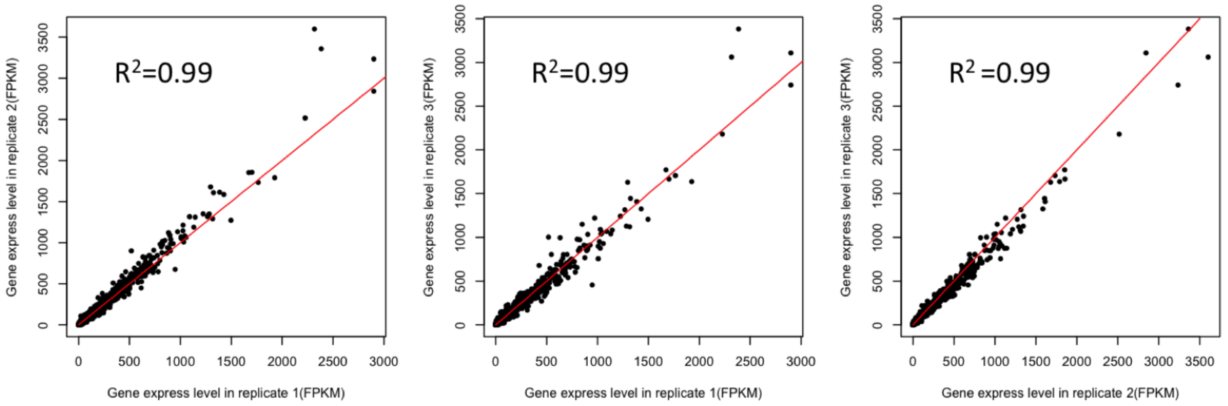

b

	Tissue	Genotype
Wild type	stage1	G
	stage4-PS	G
	stage5-PS	G
	stage5-SS	G
msd1-1	stage3	A
	stage4-PS	A
	stage4-SS	A
	stage5-PS	A
	stage5-SS	A

Supplementary Fig. 6. Quality control of transcriptome profiling of the inflorescence at different stages in WT and the *msd1-1* mutant. a) Example of high correlation among three biological replicates. The correlation coefficient was first calculated among the three replicates. After discarding two samples ($R^2 < 0.9$ relative to another sample), the average Pearson correlation coefficient of the replicates was 0.97, indicating that the remaining samples were of high quality. **b)** The *MSD1* gene genotype of the *msd1-1* mutant was confirmed in the RNA-seq data.

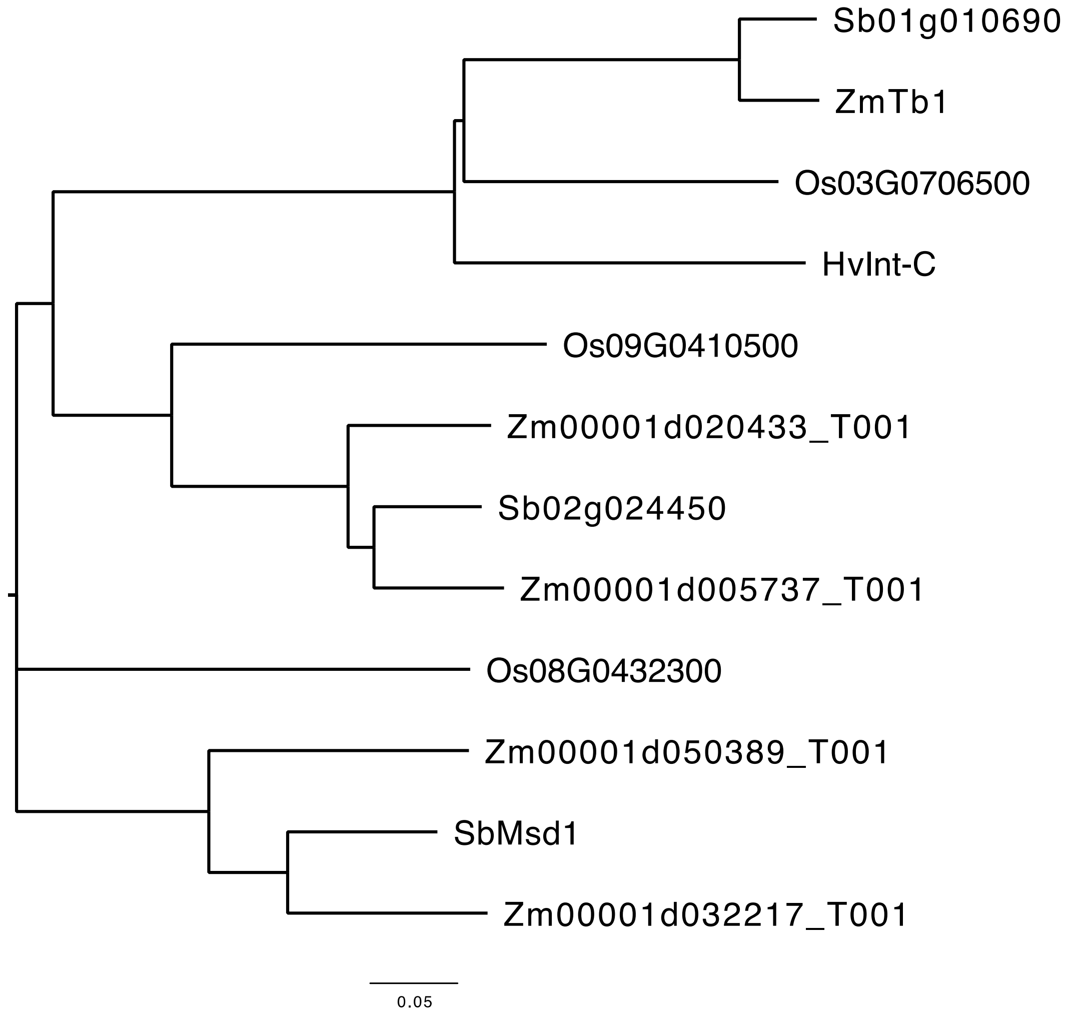

Supplementary Fig. 7. Neighbor-joining tree of maize *TB1*, sorghum *MSD1*, Barley *Int-C* gene and other TCP transcriptional factors of the CYC/TB1 subgroup in maize (Zm), sorghum (Sb), and rice (Os).

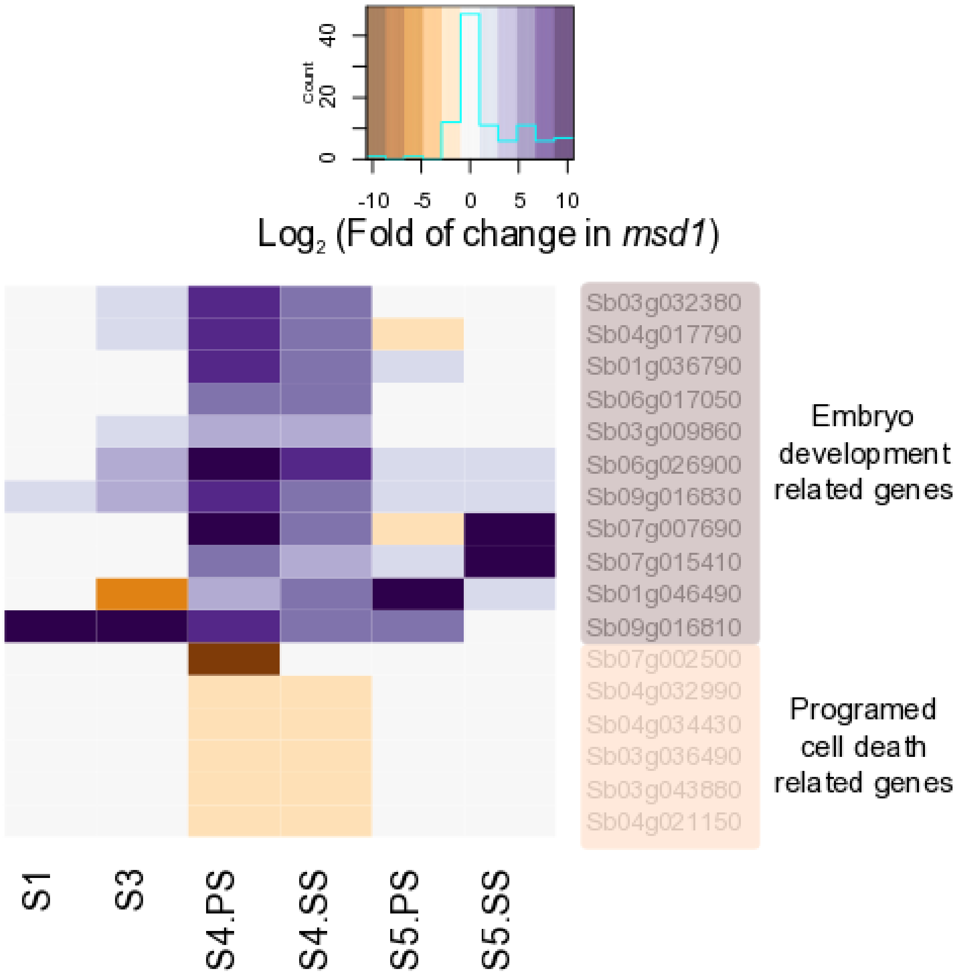

Supplementary Fig. 8. Changes in the expression of genes involved in embryo development and programmed cell depth. The colors represent (log_2 [FPKM in *msd1*] / [FPKM in WT]).

Supplementary Table 1. Complementation analysis of *msd1* alleles.

	msd1-1	***msd1-2***	***msd1-3***	***msd1-4***	***msd1-5***	***msd1-6***	***msd1-7***
msd1-1							
msd1-2	*msd*						
msd1-3	*msd*						
msd1-4	*msd*		*msd*				
msd1-5	*msd*			*msd*			
msd1-6	*msd*		*msd*		*msd*		
msdd1-7	*msd*		*msd*	*msd*		*msd*	

**msd1-1* and *msd1-2* were known to be allelic before the *MSD1* gene was identified. All F1 plants from crosses of *msd1-1* to newly identified *msd1* alleles discovered by sequencing of *msd* mutants exhibited the *msd1* phenotype. A few crosses between the new alleles of *msd1* also exhibited the *msd* phenotype. The results of the complementation test revealed that *Sb07g021140* is the *MSD1* gene. Black squares represent self-pollination. Blank squares indicate complementation crosses that were not performed.

Supplementary Table 2. Summary of the amount of data generated by RNA-seq.

		S1: Inflorescence meristem	S3: Floral transition	S4: Floral Stage 1		S5: Floral Stage 2	
				SS	PS	SS	PS
BTx623	Replicate 1	47,687,824	41,772,776	113,694,762	129,785,826	45,137,206	101,194,034
	Replicate 2	25,577,260	65,589,362	74,831,556	97,749,120	95,511,818	69,511,744
	Replicate 3	41,773,186	44,243,680	65,393,744	76,469,852	170,454,056	64,850,906
msd1 mutant	Replicate 1	32,565,494	30,692,720	45,024,586	71,309,336	89,798,568	64,127,766
	Replicate 2	65,145,788	26,076,258	26,396,264	40,340,992	82,484,548	72,818,954
	Replicate 3	35,007,414	38,994,690	38,768,952	48,652,344	106,433,328	72,850,352

*The numbers are in bp.

Supplementary Table 3. Summary of genes differentially expressed between WT and the *msd1* mutant.

	No. of expressed gene		Differential expression gene (FDR adjusted p<0.05)*	Uniquely expressed gene (FPKM>1)	
	WT	*msd1*		WT	*msd1*
S1	21,047	21,121	1,239	33	23
S3	21,470	22,247	1,500	75	34
S4-PS	24,580	21,448	10,226	156	436
S4-SS	23,846	22,218	8,490	230	339
S5-PS	23,250	24,120	1,685	69	32
S5-SS	24,223	24,590	2,063	42	36
Total	26,654	27,218	13,527	557	997

*P value was adjusted by the FDR from the Benjamini-Hochberg correction for multiple-testing.

Supplementary Table 4. The 10 most highly up-regulated and uniquely expressed in PS in the *msd1* mutant at stage 4.

	WT (FPKM)	*msd1* (FPKM)	Fold of change	Function annotation
Sb06g026900	0.238861	371.468	1555.16	Late embryogenesis abundant protein, group 1 protein
Sb01g012640	0.171734	126.108	734.322	cupin family protein
Sb04g017790	0.305038	120.424	394.784	Seed maturation protein
Sb09g016810	0.285748	92.2867	322.965	Late embryogenesis abundant 6
Sb03g032380	0.452652	139.698	308.621	Late embryogenesis abundant protein (LEA) family protein
Sb01g036790	0.272418	41.8924	153.78	embryonic cell protein 63
Sb09g016830	2.06676	295.181	142.823	Late embryogenesis abundant 1
Sb10g003700	0.81532	115.233	141.335	dehydrin xero 1
Sb02g040650	0.26434	32.7906	124.047	1-cysteine peroxiredoxin 1
Sb01g043980	0.248705	29.2706	117.692	RmlC-like cupins superfamily protein
Sb02g022095	0	92.343	inf	
Sb05g016755	0	41.0056	inf	
Sb03g027122	0	35.2284	inf	
Sb01g009730	0	32.6082	inf	
Sb07g021917	0	31.9366	inf	
Sb03g011080	0	25.9257	inf	
Sb07g023010	0	25.1244	inf	alpha-amylase-like
Sb02g010080	0	22.7582	inf	AWPM-19-like family protein
Sb07g007690	0	22.6408	inf	Late Embryogenesis Abundant 4-5
Sb03g019411	0	20.1341	inf	

Supplementary Table 5. GO term enrichment in genes up-regulated in PS in the *msd1* mutant at stage 4.

GO term	Description	No. of up-regulated genes	No. of genes in sorghum genome	p-value	FDR
GO:0009791	post-embryonic development	965	1644	2.00E-49	3.70E-45
GO:0032501	multicellular organismal process	2037	4820	3.50E-28	3.30E-24
GO:0007275	multicellular organismal development	1875	4512	7.50E-23	4.70E-19
GO:0009987	cellular process	5750	16277	7.00E-20	3.30E-16
GO:0044085	cellular component biogenesis	628	1307	3.20E-16	1.20E-12
GO:0032502	developmental process	2052	5263	3.20E-15	1.00E-11
GO:0003006	reproductive developmental process	768	1695	4.50E-15	1.20E-11
GO:0006996	organelle organization	840	1895	1.10E-14	2.60E-11
GO:0016043	cellular component organization	1314	3205	3.40E-14	7.20E-11
GO:0065007	biological regulation	2754	7386	1.80E-13	3.00E-10
GO:0022607	cellular component assembly	410	807	1.60E-13	3.00E-10
GO:0034641	cellular nitrogen compound metabolic process	370	718	6.20E-13	9.80E-10
GO:0009886	post-embryonic morphogenesis	105	122	7.90E-13	1.20E-09
GO:0065003	macromolecular complex assembly	325	612	1.10E-12	1.40E-09
GO:0044260	cellular macromolecule metabolic process	3252	8922	2.20E-12	2.80E-09
GO:0050789	regulation of biological process	2437	6509	3.20E-12	3.80E-09
GO:0043933	macromolecular complex subunit organization	356	698	4.60E-12	5.10E-09
GO:0006461	protein complex assembly	204	342	1.30E-11	1.30E-08
GO:0070271	protein complex biogenesis	204	342	1.30E-11	1.30E-08
GO:0048856	anatomical structure development	1500	3841	4.60E-11	4.30E-08

*The analysis was done by agriGO (http://bioinfo.cau.edu.cn/agriGO/). The enrichment was calculated by the Hypergeometric test. Multi-test adjustment was performed by the Yekutieli (FDR under dependency) method.

Supplementary Table 6. Primers used in RT-PCR and *in situ* experiments.

Primer name information	GENE ID	Method	Sequence information
Sb-MSD1-RT-2127F	Sb07g021140	qRT-PCR	CTACTACCAGTATTGCCTGCAGCTC
Sb-MSD1-RT-2128R	Sb07g021140	qRT-PCR	GTACTATGTAGCAACGGATCATGG
SB_EIF4α -2279F	Sb04g003390	qRT-PCR	CAACTTTGTCACCCGCGATGA
SB_EIF4α -2280R	Sb04g003390	qRT-PCR	TCCAGAAACCTTAGCAGCCCA
Sorghum-insitu-3p-1708F	Sb07g021140	Cloning for *in situ*	GGGCTGGATCATGACGGAGGCCACAGCG
Sorghum-insitu-3p-1709R	Sb07g021140	Cloning for *in situ*	GCTAATGGGCTTTAATTGATAGAGAAA
MSD1-2087F	Sb07g021140	Cloning for tobacco transient assay	ATGCCGTCGACCGCGATGTCTTGGGACG
MSD1-2088R	Sb07g021140	Cloning for tobacco transient assay	CCTTGGCATTCCTCCGTTGCATCTCATCAT
MSD1-2089F	Sb07g021140	Cloning for tobacco transient assay	GGGGACAAGTTTGTACAAAAAAGCAGGCTCCATGCCGTCGACCGCGATGTCTTGGGACG
MSD1-2090R	Sb07g021140	Cloning for tobacco transient assay	GGGGACCACTTTGTACAAGAAAGCTGGGTGCCTTGGCATTCCTCCGTTGCATCTCATCAT
