# Supplementary material for: MSD1 regulates pedicellate spikelet fertility in sorghum through the jasmonic acid pathway
Source: Nat Commun. 2018 Feb 26;9:822. doi: 10.1038/s41467-018-03238-4 (PMC5826930; doi:10.1038/s41467-018-03238-4)
Supplement: Supplementary file 2 — Peer Review File [file 41467_2018_3238_MOESM2_ESM.pdf]

PEER REVIEW FILE

Reviewers' comments:

Reviewer #1 (Remarks to the Author):

-What are the major claims of the paper?
Authors have identified a novel TCP transcriptional regulator important for grain development of pedicellate spikelets (PS) in sorghum. In a nutshell, authors provide evidence for a new mechanism that JA levels in PS determine female-fertility of floral organs and subsequently grain set! This phenotype provides opportunities to further increase sorghum yields in the future. I herewith would like to congratulate the authors for this excellent piece of work.

-Is the work convincing, and if not, what further evidence would be required to strengthen the conclusions?
Yes, very convincing! Conclusions are all justified.

-If the conclusions are not original, it would be helpful if you could provide relevant references.
See my comment 1 below.

-Are they novel and will they be of interest to others in the community and the wider field?
These findings are of high significance for the entire grass and crop plant community.

-Please feel free to raise any further questions and concerns about the paper.

Major points.
1) Authors unfortunately missed out on citing a very relevant paper for this study dealing with "Comparative phylogenomic analysis provides insights into TCP gene functions in Sorghum" by Francis et al. 2016 published in Scientific Reports 6:38488 (DOI: 10.1038/srep38488). Clearly, this publication does not harm the novelty of the present ms; nevertheless, work performed in this paper must be recognized. For example, the present ms should be following the latest gene nomenclature for sorghum and therefore must replace old gene names (Sb07g021140) with those used in the Francis et al. paper (Sobic007G135700 and SbTCP16). Moreover, Suppl. Fig 4 is redundant and should be deleted while rather citing the Francis et al. paper in L087. Similarly, in L105-107 data is presented identical to Francis et al.; therefore, delete Suppl. Fig 6 and cite Francis et al. rather than #8,9. L107-109 nuclear localization data confirms data shown by Francis et al.; cite paper after Fig. 3k.

2) L051-052: TSM. It is not very clear for me how to recognize the TSM?! The way I understand a TSM is that ONE meristematic dome (TSM) will cleave at two sites to give rise to three independent SMs (i.e. PS, SS, PS). The domes you show is SM; where are the TSMs; so the meristem/stage prior to SM?

3) Suppl. Table 1: the allelic test table is confusing to me; why you have not msd1 phenotypes in all white boxes; if msd1 to 7 are all allelic? To show column one is fine, I guess.

4) Usage of the word “seed”. While working with sorghum, using the word “seed” seems not really appropriate. Like all grass crops, sorghum produces a caryopsis also commonly known as kernel or grain. A caryopsis is developmentally, strictly characterized by the fusion of endosperm and embryo of which only the latter could be called “seed”. I therefore strongly suggest replacing ALL events where the word “seed” has been used with either kernel or grain. Since the mutant has unfortunately been given the misleading name multi-seeded 1 (msd1) in a previous paper, I assume sticking with this name is unavoidable; though not really a correct mutant description (rather multi-grained; multi-kernel).

5) Figure 5: though I like it, I do not understand the meaning of the blue box containing msd1. Depending on the allelic status at MSD1, I can see there are two possible routes/fates for floret development. While I get the progression of steps involved in PS sterility (JA -> PCD -> suppressed embryo dev), the used symbols in the msd1 mutant are not suitable. In the msd1 mutant, I assume that JA biosynthesis is not suppressed (as the symbol suggests!); it is rather NOT activated! So, using the arrow symbol with strikethrough is more appropriate instead. The following events are also not appropriately labelled. Instead of PCD it should rather be called “Prevention of PCD” followed by induction of female-fertility in PS and embryogenesis.

6) L123-127 or/and L129-137: for those readers not so familiar with JA, I think authors are advised to introduce JA action and significance for floral development by citing a few relevant papers (reviews or similar) and may elaborate a bit more about the role of JA during floral development, PCD and sex-determination (e.g. in maize).

Minor points.
7) L026: TCP has not been defined.

8) L089: Suppl. Fig 5, I only see four arrows instead of six; please add and clarify.

9) L151-152: replace “pedicellate” with “spikelet”; barley does not have pedicellate spikelets in WT plants. Only the vrs4(HvRA2) mutant shows PS in the laterals.

10) Check legend of Suppl Fig 3; colors for stars are wrong etc…

11) Suppl Fig 9: specify for which genotype the heat map has been produced; WT or mut?

Reviewer #2 (Remarks to the Author):

Manuscript NCOMMS-17-22936-T by Jiao et al. reports the cloning and characterization of the gene corresponding to the MSD1 locus, a locus that regulates development of pedicellate spikelets (PS) in sorghum. In wild type plants, PS do not develop whereas in msd1 mutants, PS develop floral organs similar to sessile spikelets (SS). The authors discovered and previously published a paper describing msd1 mutant phenotypes that results in larger panicles that have more seed relative to wild type.

1. MSD1 gene cloning: In this report, the authors clone the MSD1 gene using standard methods of bulk segregant sequencing of small F2 families followed by validation through analysis of several msd-alleles. The gene encoded by MSD1 is a TCP transcription factor. This is a major advance in our understanding of the mechanistic basis of the multi-seed trait in sorghum. Technically, the approaches used and data presented leading to gene discovery were clear and convincing.

2. Identification of MSD1 homologs in other species: One page 4 the authors report that the TCP-factor (MSD1) is in the same subgroup as sorghum homologs of HvTB1, a gene (INTERMEDIUM-C) that modifies spikelet fertility/architecture. The data presented in S-Fig 4 identifies sorghum TB1 and shows that MSD1 and a third gene/protein are in a cluster of class II TCP factors. While the authors state that MSD1 is not an ortholog of HvTB1, the basis for this statement would be worth clarifying (one assumes that SbTB1 is the ortholog of HvTB1). The authors could report which sorghum gene is collinear with HvTB1 (INT-C) and whether there is a barley TCP gene that is collinear with MSD1. TB1 alters plant tillering, so if the authors have information on tillering in WT vs. msd1, that would also be useful to report. Panicle branching is altered in msd1, consistent with a TB1-modualted phenotype.

3. In situ hybridization of MSD1 transcript tissue x development analysis: These results are shown in Fig 3. MSD1 is shown to be differentially expressed in the spikelet, with >10-fold higher expression in PS-S4 compared to S1 (and other vegetative tissues) (S4-PS shows the highest relative expression). It would have been useful to include data on S2 in the same figure (Fig 3, I). For presentation purposes, showing the data in sub-Fig3 I (graphical expression vs. development) first, then the in situ hybridization figures (spatial) might be helpful. The in situ hybridization results include data on stages S2 and S3. Its unfortunate that in situ hybridization data on S4 (SS, PS) was not included since this is where the key difference in overall expression occurs, and where gene action is proposed to affect spikelet differentiation. A key question not

addressed is where differential expression of MSD1 occurs in PS at stage 4.

4. RNA-seq analysis: The technical approach is excellent - and the authors acknowledge that any event that disrupts PS development will result in large differences in global expression in PS. This is why the top genes with differential expression are involved in seed development (p 5). On page 5 and in the methods, the authors briefly describe how they identified genes that are differentially expressed in PS of MSD1 vs. msd1 plants, leading to their hypothesis that MSD1 specifically regulates JA biosynthesis (vs. the many other pathways that are differentially expressed). The approach used could be clarified, and if possible, further validated by reporting all pathways that are differentially expressed during PS development in WT vs. msd1 plants, and how all but JA synthesis/signaling are eliminated using their approach.

Fig 4 shows that Msd1 and genes involved in JA synthesis and signaling are differentially expressed in PS vs. SS at stage 4 in the WT (Fig 4a). MSD1 in the WT is expressed at similar levels in SS at stage 3 and 4. However, genes involved in JA are strongly up-regulated. I guess the authors would explain this by suggesting that JA is regulated by several factors including MSD1. MSD1 is induced 4x in PS between stage 3 and 4, but AOS (Sb01g007000) is induced ~100x. Again, if MSD1 and other factors combine to induce AOS (and other genes involved in JA synthesis), then proportional induction is not expected. The key difference the authors point out is that AOS and other genes in the JA-synthesis/signaling pathway are not induced to the same extent in PS of msd1 plants compared to WT (AOE looks like a 50% reduction) and that differential expression between PS and SS observed in WT is not observed. Of course this is expected since SS and PS organs in msd1 plants develop normally in parallel. Also, since PS in WT is induced to undergo PCD, expression of genes involved in JA could be reduced relative to msd1 because PS organs are undergoing different developmental events.

One suggestion to further support the author's hypothesis is to do a co-expression analysis to see which genes are expressed in a manner consistent with regulation by MSD1, and that are differentially expression in comparisons of MSD1 and msd1, specifically during development of PS. I realize that co-expression analysis will be confounded by the large number of genes that are differentially expressed during PS development, so RNA-seq analysis at the onset of MSD1 vs. msd1 impact on PS development may be required and also difficult to obtain.

5. JA levels and Me-JA rescue: The authors show that JA levels in panicles of WT are 2-3X higher than panicles of msd1 mutants. Since the panicles are different size and contain different numbers of seed (or nascent spikelets?), it would be helpful to determine if JA levels in PS or SS are different. Also, if the authors obtained RNA-seq data on PS from msd1 plants treated JA, it would be interesting to know if the PS profiles are similar to WT as expected (or did the JA treatment phenocopy the WT through a different mechanism).

Me-JA rescue was done by treating developing panicles in situ, followed by analysis of the emerged panicle. The results in Fig 4d look convincing. It might be worth including statistical data to further support this photograph (unless this was already provided in a supplemental figure).

Did the authors measure JA levels in panicles of plants treated with Me-JA? Was the rescue done with a physiological amount of JA (1 mM is high relative to endogenous levels shown in Fig 4c, however, only a small portion of the JA probably reached the panicle).

Did the JA that restores WT PS development also induce genes involved in PCD?

6. Model in Figure 5 and other speculations: Figure 5 is logical, but perhaps not too informative and could be eliminated. Clearly, MSD1 is a key regulator of PS development, therefore any process associated with the divergent developmental pathways (PCD, LEA expression) will be downstream of MSD1. I would recommend that the authors focus on the key finding (hypothesis) that MSD1 regulates JA synthesis/signaling which leads to inhibition of PS development.

Discussion: The authors suggest that the mechanism involved in MSD1 mediated PS developmental cessation involves JA and that this is different from that modulated by barley VRS1 which was shown to modulate sugar, auxin, CK and GA gradients. Since the study of Youssef et al did not include JA, and the current study of MSD1 did not include analysis of sugars, IAA, CK, and GA, it might be best to just note that the mechanisms could be different, but that will require additional testing.

The authors may also want to discuss the results of Talk and Schwartz (2017) showing that the multi-seed trait did not increase yield or harvest index but instead decreased yield.

The authors may also consider discussing results reported by Acosta et al (2009) tasselseed1 is a lipoxygenase affecting JA signaling in sex determination of Maize. That paper described the role of Ts1, a gene encoding a lipoxygenase involved in JA biosynthesis, that mediates PCD of pistil primordia in staminate florets. In fact they used a similar JA rescue (same concentration). This citation would support the authors model.

Overall, the data presented in this paper will be of interest to researchers working on plant development and those interested in increasing grain yield.

Reviewer: John Mullet

Reviewer #3 (Remarks to the Author):

In this work, Jiao et al characterize the sorghum mutant msd1, which is able to set seed in both pedicellate and sessile spikelets (PS and SS), in contrast with wild-type sorghum, where only SS are fertile:

1) The authors describe the developmental features of wild-type and msd1 inflorescences and determine that floral organs abort by stage 5 in wild-type PS while they continue to develop in msd1.

2) They convincingly identify the Msd1 gene as encoding a TCP transcription factor.

3) They attempt to establish the in situ spatiotemporal dynamics of Msd1 gene expression.

4) By RNAseq analysis, they identify jasmonate signalling genes as potential targets of Msd1 transcriptional activation.

5) They show that jasmonate levels are reduced in the msd1 mutant and that external application of jasmonate to msd1 inflorescences recapitulates the wild-type phenotype.

Overall, these are exciting findings that confer a new putative function for the plant hormone jasmonate in cereal inflorescence development. They also provide further, more concrete support to the proposed regulation of jasmonate synthesis/signalling by TCP transcription factors. The work also has important implications for the long-term goal of increasing cereal seed yield.

The main claims of the research are well supported by the experimental evidence, which is in general clear, well presented and it seems derived from rigorous experiments.

Still, there are several aspects that could be improved to further strengthen the work, as follows:

Major issues

1. My main concern is about the Msd1 in situ hybridization data. I was particularly surprised that Fig. 3 only displays Msd1 in situ expression for sections of inflorescences at stages 2 and 3. I believe that it is vital to assess Msd1 in situ expression at stage 4 because at such stage: a) the developmental arrest of floral organs is seen; b) Msd1 expression reaches its peak according to

Fig. 4a and Suppl. Fig. 8. Furthermore, the authors themselves propose in their predicted expression pattern for Msd1 target genes and in their final model (lines 141-142) that Msd1 expression is activated in PS at that stage, implying that it is the most important for Msd1 function. In addition to this temporal aspect of Msd1 expression, I believe that it is equally important to better assess the spatial distribution of Msd1 to confirm the authors' observation by qPCR and RNAseq that there is an obvious difference in Msd1 expression between PS and SS at stage 4. I would expect to see in situ data for both spikelet types at stage 4.

2. More regarding the in situ expression data, lines 100-102: "Expression was maintained within floral organ primordia until the floral meristem initiated, and then expanded to the tip of floral meristem, including the glume, at stage 3". I thought that floral organ primordia produce floral organs; and that floral meristems are the ones that initiate flowers. So, I believe that floral meristems should precede floral organ primordia, should not they? The author's statement implies that floral organ primordia appear before floral meristem. Are they using the wrong terminology here or I am missing something?

3. In general, the description of the in situ hybridization patterns could benefit of a clearer presentation/explanation.

4. I do not understand how Supplemental Figure 4 supports that MSD1 is "a class II TCP transcription factor belonging to the CYC/TB1 subgroup" (lines 86-87 of the text). That figure is just a phylogeny and alignment of TCPs within sorghum. Are there any 'defining' features in the alignment that support that classification? Or is the classification supported by MSD1's homology to specific orthologs in other species? If the later, then those orthologs have to be included in the phylogeny and the defining features of the subgroup clarified in the alignment.

5. Additionally, why limit the phylogenetic analysis to the grasses in Suppl. Fig. 5? Are the TCPs too different in the dicots? I think it would be more interesting and informative to know if the amino acids affected in the msd1 mutant allelic series are also conserved (and presumably important) in other orthologous TCPs beyond the grasses. In sum, I believe Suppl. Figures 4 and 5 could be made into a single figure with a proper phylogeny and alignment containing less grasses and paralogous sequences and more orthologous sequences beyond the grasses, from the dicots if possible.

6. It would also be useful to mark the location of barley INTERMEDIUM C in the phylogenetic tree in Supplemental Figure 5, to have an idea of how close to MSD1 is.

7. Lines 121-122: I believe that at this point the authors need to define better what an 'Msd1 target gene' is. The method section entitled "Identification of putative regulatory targets of Msd1" should be part of the main text here because it is important information to understand the

results presented in Figure 4.

8. Even more importantly: The pattern of expression of JA-related genes should be explained/emphasized in the main text (somewhere between lines122-127). Only in the legend to figure 4, the authors state that “Genes involved in biosynthesis were down-regulated at stage 4 in PS of msd1”. This information is very important evidence (and one of the central findings of this work), so it should be explicitly stated in the text, not just in a figure legend. In lines 125-127 the authors simply state that JA biosynthesis genes follow “a pattern consistent with Msd1 regulation”. However, as stated above, the explanation of what this “pattern” means was left down in the methods and it is not clear/obvious where it matters in the main text.

9. Lines 129-130. “…we measured the changes induced by JA”. I believe the authors measured “the changes in JA levels”, not any changes induced by JA.

10. Lines 134-137: “We also noted that prolonged methyl-JA treatments at concentrations greater than 1 mM decreased overall panicle size and branching compared to controls, consistent with the observation that msd1 mutants tended to have much larger panicles than WT from our previous study”. With this long statement, the authors seem to imply a relationship between JA levels and panicle size, based on the observation of smaller panicles with higher-than-1mM JA treatments. However, in the method section, lines 335-336 the authors clearly state that higher-than-1mM JA concentrations “severely retarded growth and development of the plant”. Therefore, I do not think any conclusions should/can be drawn from such high concentration treatments.

11. Lines 142-144. I think it is relevant to cite Acosta et al, 2009 (Science, 323: 262-5. PubmedID: 19131630), which more specifically suggests a role for jasmonate in flower organ abortion also probably via programmed cell death.

12. Line 148. I fail to see how figure 4a supports the statement about high expression of embryogenesis-related genes in the msd1 mutant.

13. Supplementary Figure 8. It would be preferable to plot the qRT-PCR data in the same fashion as in Figure 4a. It is so much harder to read the data with yet a different plot display! Additionally, the plots in Suppl. Figure 8 create the false idea of developmental time in the x-axis, while in reality two different tissue types (PS and SS) are mixed together. Additionally: a) What are the samples S1 and S3 in that experiment? Only PS or only SS or a mix of both at that stage? b) Can the authors provide an explanation to the differences in gene expression quantified by RNAseq vs. qRT-PCR? For example, in Figure 4a Msd1 expression in stage 4 msd1 mutant SS is very low relative to the PS of both wild-type and mutant. However, in suppl. Fig. 8, stage 4 msd1 mutant SS displays higher Msd1 expression than mutant stage 4 PS.

14. Line 155. "… by de-repression of PS development in sorghum panicles through blockage of JA-induced programmed cell death". This is an overstatement: No evidence indicating that JA is promoting cell death is presented in this manuscript. Additionally, the involvement of cell death is only circumstantial (from the RNAseq data); further evidence supporting a causative role of cell death in PS suppression is not presented. Therefore, the text in line 155 should probably read something like"…by de-repression of PS development in sorghum panicles through blockage of JA signalling and possibly programmed cell death".

15. It may be worth including a line or two in the discussion to cite Schommer et al, 2008 (PLoSi Biol.6(9):e230. PubmedID: 18816164). That work was the first to suggest a role for class II TCP transcription factors in promoting JA biosynthesis.

16. Another reference that may need citation is Cai et al, 2014 (https://www.nature.com/articles/ncomms4476), which reported that JA is required for early spikelet development in rice (flower meristem determinacy and floral organ identity). This is in contrast to the negative role of JA later in floral organ development reported here by Jiao et al.

17. In the legend to figures 1j-m, there is no clarification about the inflorescence stage that is displayed.

Minor issues

18. In figure 1j-m, are the black lines 'surrounding' the ovary actually part of the tissue structure? Or were they added 'artificially' to emphasize the contour of the carpel? Please explain in the legend.

19. In the 'pipeline' graph of figure 2a, one panel seems repeated; or is that step actually done twice in the actual pipeline?

20. The legend to Suppl. Fig. 3 seems incorrect (panel labelling does not correspond to legend).

21. The figure legend to Supplemental Figure 5 is incomplete

22. Line 59: "...to develop…" should/can be erased.

23. Line 114, incorrect citation, not supplementary figure 4.

24. Line 116. "…the identities of the genes". I think the authors mean "the number of the genes".

Responses to Reviewers:

Reviewer #1 (Remarks to the Author):

-What are the major claims of the paper?
Authors have identified a novel TCP transcriptional regulator important for grain development of pedicellate spikelets (PS) in sorghum. In a nutshell, authors provide evidence for a new mechanism that JA levels in PS determine female-fertility of floral organs and subsequently grain set! This phenotype provides opportunities to further increase sorghum yields in the future. I herewith would like to congratulate the authors for this excellent piece of work.

Thank you!

-Is the work convincing, and if not, what further evidence would be required to strengthen the conclusions?
Yes, very convincing! Conclusions are all justified.

-If the conclusions are not original, it would be helpful if you could provide relevant references.
See my comment 1 below.

-Are they novel and will they be of interest to others in the community and the wider field?
These findings are of high significance for the entire grass and crop plant community.

-Please feel free to raise any further questions and concerns about the paper.

Major points.
1) Authors unfortunately missed out on citing a very relevant paper for this study dealing with "Comparative phylogenomic analysis provides insights into TCP gene functions in Sorghum" by Francis et al. 2016 published in Scientific Reports 6:38488 (DOI: 10.1038/srep38488). Clearly, this publication does not harm the novelty of the present ms; nevertheless, work performed in this paper must be recognized. For example, the present ms should be following the latest gene nomenclature for sorghum and therefore must replace old gene names (Sb07g021140) with those used in the Francis et al. paper (Sobic007G135700 and SbTCP16). Moreover, Suppl. Fig 4 is redundant and should be deleted while rather citing the Francis et al. paper in L087. Similarly, in L105-107 data is presented identical to Francis et al.; therefore, delete Suppl. Fig 6 and cite Francis et al. rather than #8,9. L107-109 nuclear localization data confirms data shown by Francis et al.; cite paper after Fig. 3k.

Thank you for these suggestions. Francis et al (2016) is cited in the revised manuscript as suggested. In addition, we made the following revisions: 1) *SbTCP16* is used to refer to the *Msd1* gene; and 2) Suppl. Fig 4, Fig 5a, and Fig. 6 were eliminated. We retained the version 1 gene ID because we started our project before the new nomenclature for the second and third versions of

the sorghum reference genome was available. To change the nomenclature of the gene ID would require us to redo of all the data analysis for this paper, which would not be possible at this point. The whole study, as well as a patent (https://www.google.com/patents/US20160264986), was based on the sorghum version 1 reference genome. After careful checking, we concluded that there was no change in gene sequence among the three versions of sorghum reference genome. The different versions of the gene ID are also still searchable in both the Gramene and Phytozome databases. To clarify, we now state that the data analysis was performed using the version 1 sorghum reference genome.

2) L051-052: TSM. It is not very clear for me how to recognize the TSM?! The way I understand a TSM is that ONE meristematic dome (TSM) will cleave at two sites to give rise to three independent SMs (i.e. PS, SS, PS). The domes you show is SM; where are the TSMs; so the meristem/stage prior to SM?

Yes, TSM is a single meristematic dome that arises prior to SM. Thus, the SM appears after TSM during development. Each spikelet meristem contains PS and SS. In the supplementary Figure 2, we added TSM text in the supplementary Figure 2a with blue color asterisks at the stage 1 and also change the figure legend of supplementary figure2. And, you can see each SM in supplementary figure 2a with red color asterisks.

3) Suppl. Table 1: the allelic test table is confusing to me; why you have not msd1 phenotypes in all white boxes; if msd1 to 7 are all allelic? To show column one is fine, I guess.

We performed allelic tests between *msd1-1* and all other alleles, which should be sufficient to determine allelism. We also performed some crosses between *msd1* alleles before we knew they were allelic, but these crosses were not complete. All the other crosses we performed resulted in no complementation, so we did not conduct additional complementation crosses of these mutants after we found out they were allelic to *msd1-1*. The blanks in this table indicate complementation crosses we did not perform, and the table legend was updated to make this clear.

4) Usage of the word "seed". While working with sorghum, using the word "seed" seems not really appropriate. Like all grass crops, sorghum produces a caryopsis also commonly known as kernel or grain. A caryopsis is developmentally, strictly characterized by the fusion of endosperm and embryo of which only the latter could be called "seed". I therefore strongly suggest replacing ALL events where the word "seed" has been used with either kernel or grain. Since the mutant has unfortunately been given the misleading name multi-seeded 1 (msd1) in a previous paper, I assume sticking with this name is unavoidable; though not really a correct mutant description (rather multi-grained; multi-kernel).

As mentioned, the mutants were named in the previous publication and patent, so we changed "seed" to "grain" in the text, but retained "*msd*" as the mutant symbol as suggested.

5) Figure 5: though I like it, I do not understand the meaning of the blue box containing msd1. Depending on the allelic status at MSD1, I can see there are two possible routes/fates for floret development. While I get the progression of steps involved in PS sterility (JA -> PCD -> suppressed embryo dev), the used symbols in the msd1 mutant are not suitable. In the msd1 mutant, I assume that JA biosynthesis is not suppressed (as the symbol suggests!); it is rather NOT activated! So, using the arrow symbol with strikethrough is more appropriate instead. The following events are also not appropriately labeled. Instead of PCD it should rather be called "Prevention of PCD" followed by induction of female-fertility in PS and embryogenesis.

The blue box indicates the expression pattern of MSD1 during panicle development. MSD1 expression was detected in stage 1, increased through stage 3 peaked in stage 4, and decreased in stage 5.

As pointed out by the reviewers, we do not have strong evidence to support direct regulatory links with PCD and LEA genes, so we eliminated the model in the original Figure 5. Instead of a figure, we describe our hypothetical regulatory model in the Discussion section on page 10.

6) L123-127 or/and L129-137: for those readers not so familiar with JA, I think authors are advised to introduce JA action and significance for floral development by citing a few relevant papers (reviews or similar) and may elaborate a bit more about the role of JA during floral development, PCD and sex-determination (e.g. in maize).

We agree that some background about JA would help readers who are not already familiar with it. Accordingly, we added a new paragraph introducing the JA pathway and its known functions in plant development to the Introduction on page 3.

Minor points.

7) L026: TCP has not been defined.

In this revised version, we define TCP as (Teosinte branched/Cycloidea/PCF) in the Abstract.

8) L089: Suppl. Fig 5, I only see four arrows instead of six; please add and clarify.

A: Thank you for pointing this out. In the original Suppl. Fig 5b, only the five amino acid changes were pointed out. The first two SNPs *(msd1-1 and msd1-2)* changed the same amino acid, and the mutation in *msd1-6* is a stop-codon gain, which is not shown in the original Suppl.

Fig5. As a result, there should be a total of five arrows. To make the figure easier to read, we changed the color of the arrows from black to red in the current Supplementary Fig. 4.

9) L151-152: replace "pedicellate" with "spikelet"; barley does not have pedicellate spikelets in WT plants. Only the vrs4(HvRA2) mutant shows PS in the laterals.

The suggested revision has been made.

10) Check legend of Suppl Fig 3; colors for stars are wrong etc…
A: Thank you for pointing out the error. We have corrected the order of this figure.

11) Suppl Fig 9: specify for which genotype the heat map has been produced; WT or mut?
A: Suppl Fig 9 (now updated as Suppl. Fig 8) shows the fold change in gene expression in the *msd1* mutant vs. WT. The legend has been updated to make this clear.

Reviewer #2 (Remarks to the Author):

Manuscript NCOMMS-17-22936-T by Jiao et al. reports the cloning and characterization of the gene corresponding to the MSD1 locus, a locus that regulates development of pedicellate spikelets (PS) in sorghum. In wild type plants, PS do not develop whereas in msd1 mutants, PS develop floral organs similar to sessile spikelets (SS). The authors discovered and previously published a paper describing msd1 mutant phenotypes that results in larger panicles that have more seed relative to wild type.

1. MSD1 gene cloning: In this report, the authors clone the MSD1 gene using standard methods of bulk segregant sequencing of small F2 families followed by validation through analysis of several msd-alleles. The gene encoded by MSD1 is a TCP transcription factor. This is a major advance in our understanding of the mechanistic basis of the multi-seed trait in sorghum. Technically, the approaches used and data presented leading to gene discovery were clear and convincing.

Thank you for these comments!

2. Identification of MSD1 homologs in other species: One page 4 the authors report that the TCP-factor (MSD1) is in the same subgroup as sorghum homologs of HvTB1, a gene (INTERMEDIUM-C) that modifies spikelet fertility/architecture. The data presented in S-Fig 4 identifies sorghum TB1 and shows that MSD1 and a third gene/protein are in a cluster of class II TCP factors. While the authors state that MSD1 is not an ortholog of HvTB1, the basis for this statement would be worth clarifying (one assumes that SbTB1 is the ortholog of HvTB1). The authors could report which sorghum gene is collinear with HvTB1 (INT-C) and whether there is a barley TCP gene that is collinear with MSD1. TB1 alters plant tillering, so if the authors have information on tillering in WT vs. msd1, that would also be useful to report. Panicle branching is altered in msd1, consistent with a TB1-modualted phenotype.

The old Suppl. Fig. 4 was excluded from the revised version to avoid redundancy with published Francis et al. (2016). The barley *INTERMEDIUM C* (*INT-C*) gene belongs to the same subgroup as *Msd1* (TCP class II, CYC/TB1 subgroup). There are three genes in this group in sorghum. The *Msd1* gene is not an orthology of maize *Tb1*. Thus, sorghum *Msd1* and barley *INT-C* are not from the same homology group, and the barley *INT-C* gene is the ortholog of sorghum *Tb1*: *Sb01g010690*, not *Msd1*. The text in the manuscript has also been rephrased to make this clearer. A new Supplementary Fig. 7 was also added to show this information.

3. In situ hybridization of MSD1 transcript tissue x development analysis: These results are shown in Fig 3. MSD1 is shown to be differentially expressed in the spikelet, with >10-fold higher expression in PS-S4 compared to S1 (and other vegetative tissues) (S4-PS shows the

highest relative expression). It would have been useful to include data on S2 in the same figure (Fig 3, I). For presentation purposes, showing the data in sub-Fig3 I (graphical expression vs. development) first, then the in situ hybridization figures (spatial) might be helpful. The in situ hybridization results include data on stages S2 and S3. Its unfortunate that in situ hybridization data on S4 (SS, PS) was not included since this is where the key difference in overall expression occurs, and where gene action is proposed to affect spikelet differentiation. A key question not addressed is where differential expression of MSD1 occurs in PS at stage 4.

This is good point. Stages 1 and 2 are difficult to separate. Therefore, to be more accurate, "stage 1" in Figure3 was renamed as "stage 1 and stage 2" in the revised version. In addition, as suggested, we modified Figure 3 to present the RT-PCR result first, followed by images of *in situ* hybridizations.

We now provide the stage 4 *in situ* hybridization as supplementary Figure 5. At stage 4, expression of *Msd1* was detectable, but low, in the ovaries of both SS and PS, and the highest expression was detected in anthers. JA plays a major role in pollen development or shedding in *Arabidopsis* (Stintzi, A. and J. Browse. 2000). The *Arabidopsis* male-sterile mutant, *opr3*, lacks the 12-oxophytodienoic acid reductase required for jasmonate synthesis (*Proc Natl Acad Sci U S A* 97: 10625-10630). JA may play similar roles in sorghum, but this question is beyond the reach of our data.

4. RNA-seq analysis: The technical approach is excellent - and the authors acknowledge that any event that disrupts PS development will result in large differences in global expression in PS. This is why the top genes with differential expression are involved in seed development (p 5). On page 5 and in the methods, the authors briefly describe how they identified genes that are differentially expressed in PS of MSD1 vs. msd1 plants, leading to their hypothesis that MSD1 specifically regulates JA biosynthesis (vs. the many other pathways that are differentially expressed). The approach used could be clarified, and if possible, further validated by reporting all pathways that are differentially expressed during PS development in WT vs. msd1 plants, and how all but JA synthesis/signaling are eliminated using their approach.

We identified the JA pathway as the potential regulatory target of *Msd1*, based not only on the differentially expressed gene in PS, but also on a pattern consisting of three criteria. The details of how we identified the potential regulatory targets of *Msd1* were originally presented in the Methods section: “1) Because the target genes are mainly required at stage 4 to determine the fate of PS, their expression should peak at S4 in PS in WT plants; 2) because MSD1 suppresses the development of PS in WT but not in *msd1*, the target genes should be expressed at higher levels in PS than SS in WT, but their expression levels in PS should be greatly reduced in *msd1*; and 3) because both PS and SS developed into grains in the mutant, the target genes should be expressed at similar levels in PS and SS during stage 4 in *msd1*.” In this version, to clarify, we moved this information to the Results on page 7.

Fig 4 shows that Msd1 and genes involved in JA synthesis and signaling are differentially expressed in PS vs. SS at stage 4 in the WT (Fig 4a). MSD1 in the WT is expressed at similar levels in SS at stage 3 and 4. However, genes involved in JA are strongly up-regulated. I guess the authors would explain this by suggesting that JA is regulated by several factors including MSD1. MSD1 is induced 4x in PS between stage 3 and 4, but AOS (Sb01g007000) is induced ~100x. Again, if MSD1 and other factors combine to induce AOS (and other genes involved in JA synthesis), then proportional induction is not expected. The key difference the authors point out is that AOS and other genes in the JA-synthesis/signaling pathway are not induced to the same extent in PS of msd1 plants compared to WT (AOE looks like a 50% reduction) and that differential expression between PS and SS observed in WT is not observed. Of course this is expected since SS and PS organs in msd1 plants develop
normally in parallel. Also, since PS in WT is induced to undergo PCD, expression of genes involved in JA could be reduced relative to msd1 because PS organs are undergoing different developmental events.

We agree that JA is regulated by several factors, including MSD1, so JA synthesis and/signaling pathway genes are not induced in the similar proportion in PS of *msd1* compared to wild type.

MSD1 is transcription factor that affect the expression of downstream target genes. The direct targets of MSD1 have not yet been identified, but MSD1 in combination with other factors, either TCPs or members of other TF families, can regulate *AOS* and *LOX3*. The confusion here may be also caused by the simplified description of how we generated our hypothesis about the putative regulatory targets of MSD1. This hypothesis was based on the pattern of expression change from stage 1 to stage 5 in the mutant and WT.

Instead of a figure, we present our hypothetical regulatory model in the Discussion on page 10.

One suggestion to further support the author's hypothesis is to do a co-expression analysis to see which genes are expressed in a manner consistent with regulation by MSD1, and that are differentially expression in comparisons of MSD1 and msd1, specifically during development of PS. I realize that co-expression analysis will be confounded by the large number of genes that are differentially expressed during PS development, so RNA-seq analysis at the onset of MSD1 vs. msd1 impact on PS development may be required and also difficult to obtain.

Initially, we considered conducting co-expression analysis, but we concluded that this approach would not be well suited for our goal, i.e., explore the putative regulatory targets of MSD1. In addition, we did not have enough samples to conduct co-expression analysis; normally, at least 20 samples are required for this purpose (Van Dam *et al*., Briefings in Bioinformatics, 2017).

As an alternative, we designed the pattern to identify possible regulatory targets based on the reported functions of other TCP members and the mutant phenotype. This approach revealed

enrichment of genes involved in the JA pathway, which was subsequently validated by the JA rescue experiment. More details have been added to that section on Page 7.

5. JA levels and Me-JA rescue: The authors show that JA levels in panicles of WT are 2-3X higher than panicles of msd1 mutants. Since the panicles are different size and contain different numbers of seed (or nascent spikelets?), it would be helpful to determine if JA levels in PS or SS are different.

To compare the JA level between *msd1* mutants and WT, we normalized against panicle weight in five independent replicates; the data are expressed as ng/g. We also updated this information in the Methods and Figure Legends.

At stage 4 in BTx623, PS is only ~0.3 mg (fresh weight), whereas while SS is 1.7 mg. Consequently, it was hard to collect enough tissue to conduct the measurement. The fates of floral organs were already determined before stage 4. Accordingly, the JA rescue experiment must be performed prior to stage 3. When JA was applied after stage 3, no rescue was ever observed. It is technically challenging to collect enough PS tissues for the JA assay at stage 4, and much harder for stages early than 4.

Also, if the authors obtained RNA-seq data on PS from msd1 plants treated JA, it would be interesting to know if the PS profiles are similar to WT as expected (or did the JA treatment phenocopy the WT through a different mechanism).

We agree that it would be useful to have RNA-seq data from JA-treated plants. Similar data was published for *Arabidopsis* (Hickmen et al., *The Plant Cell*, 2017 http://www.plantcell.org/content/early/2017/08/21/tpc.16.00958).
Currently, however, we cannot obtain these data because of the time required to finish this work. The RNA-seq data we have so far were collected from the field, and we would not be able to generate this new data until late next summer after performing the JA treatment and collecting sufficient tissue for the experiments.

Did the authors measure JA levels in panicles of plants treated with Me-JA? Was the rescue done with a physiological amount of JA (1 mM is high relative to endogenous levels shown in Fig 4c, however, only a small portion of the JA probably reached the panicle). Did the JA that restores WT PS development also induce genes involved in PCD?

We did not collect tissues during the JA rescue experiment because we were waiting until the plants set seeds to observe the phenotype change. Therefore, we could not measure JA level or the expression of PCD genes. A concentration of 1 mM is probably much higher than physiological level, but this concentration was used in maize to rescue the *ts1* and *opr7/opr8* mutants (Science 323:262; Plant Cell 24:1420).

6. Model in Figure 5 and other speculations: Figure 5 is logical, but perhaps not too informative and could be eliminated. Clearly, MSD1 is a key regulator of PS development, therefore any process associated with the divergent developmental pathways (PCD, LEA expression) will be downstream of MSD1. I would recommend that the authors focus on the key finding (hypothesis) that MSD1 regulates JA synthesis/signaling which leads to inhibition of PS development.

Thank you for the comments. From the expression data, LEA genes are the most highly up-regulated group at stage 4 in PS in the *msd1* mutant. Based on similar suggestions from the other two reviewers that our evidence about the Msd1–JA–PCD pathway was not direct, but was supported by the work on *Ts1* (Science 323:262), we decided to eliminate this figure. Instead, we describe our hypothetical regulatory model in the Discussion section on page 10.

Discussion: The authors suggest that the mechanism involved in MSD1 mediated PS developmental cessation involves JA and that this is different from that modulated by barley VRS1 which was shown to modulate sugar, auxin, CK and GA gradients. Since the study of Youssef et al did not include JA, and the current study of MSD1 did not include analysis of sugars, IAA, CK, and GA, it might be best to just note that the mechanisms could be different, but that will require additional testing.

We agree. Because at this point we do not have the testing data for IAA, CK, and GA, we added additional discussion.

The authors may also want to discuss the results of Talk and Schwartz (2017) showing that the multi-seed trait did not increase yield or harvest index but instead decreased yield.

The primary *msd1* mutants, without backcrossing, were used in Talk and Schwartz's work. The average mutation rate in the primary mutants is ~7800 per line (Jiao et al., *The Plant Cell* 2016). The work we reported in *Crop Science* compared F2 segregants with either msd1 or WT panicles. Thus, the effect of background mutations was largely cancelled out. Given this mutational load, it is not surprising that the *msd1* mutants had lower grain yields than WT. Even with the heavy mutational load, Talk and Schwartz confirmed our observation that the *msd1* mutants produced more grains per panicle. Furthermore, BTx623 has a strong response to shade and low tolerance to planting density. We communicated our concerns about their results, and offered to do another year's testing with mutant seeds that have been backcrossed twice. Nevertheless, they decided to publish because Talk was retiring and she wanted to wrap up her work. As such, we would not to discuss this work in this manuscript. At the same, we are also performing more accurate yield test of *msd1* phenotype under different background, which will take time to finish.

The authors may also consider discussing results reported by Acosta et al (2009) tasselseed1 is a lipoxygenase affecting JA signaling in sex determination of Maize. That paper described the role of Ts1, a gene encoding a lipoxygenase involved in JA biosynthesis, that mediates PCD of pistil primordia in staminate florets. In fact they used a similar JA rescue (same concentration). This citation would support the authors model.

We agree. This paper is discussed on page 10 of our revised manuscript.

Overall, the data presented in this paper will be of interest to researchers working on plant development and those interested in increasing grain yield.

Reviewer: John Mullet

Reviewer #3 (Remarks to the Author):

In this work, Jiao et al characterize the sorghum mutant msd1, which is able to set seed in both pedicellate and sessile spikelets (PS and SS), in contrast with wild-type sorghum, where only SS are fertile:

1) The authors describe the developmental features of wild-type and msd1 inflorescences and determine that floral organs abort by stage 5 in wild-type PS while they continue to develop in msd1.

2) They convincingly identify the Msd1 gene as encoding a TCP transcription factor.

3) They attempt to establish the in situ spatiotemporal dynamics of Msd1 gene expression.

4) By RNA-seq analysis, they identify jasmonate signaling genes as potential targets of Msd1 transcriptional activation.

5) They show that jasmonate levels are reduced in the msd1 mutant and that external application of jasmonate to msd1 inflorescences recapitulates the wild-type phenotype.

Overall, these are exciting findings that confer a new putative function for the plant hormone jasmonate in cereal inflorescence development. They also provide further, more concrete support to the proposed regulation of jasmonate synthesis/signaling by TCP transcription factors. The work also has important implications for the long-term goal of increasing cereal seed yield.

The main claims of the research are well supported by the experimental evidence, which is in general clear, well presented and it seems derived from rigorous experiments.

Thank you!

Still, there are several aspects that could be improved to further strengthen the work, as follows:

Major issues

1. My main concern is about the Msd1 in situ hybridization data. I was particularly surprised that Fig. 3 only displays Msd1 in situ expression for sections of inflorescences at stages 2 and 3. I believe that it is vital to assess Msd1 in situ expression at stage 4 because at such stage: a) the developmental arrest of floral organs is seen; b) Msd1 expression reaches its peak according to Fig. 4a and Suppl. Fig. 8. Furthermore, the authors themselves propose in their predicted expression pattern for Msd1 target genes and in their final model (lines 141-142) that Msd1

expression is activated in PS at that stage, implying that it is the most important for Msd1 function. In addition to this temporal aspect of Msd1 expression, I believe that it is equally important to better assess the spatial distribution of Msd1 to confirm the authors' observation by qPCR and RNA-seq that there is an obvious difference in Msd1 expression between PS and SS at stage 4. I would expect to see in situ data for both spikelet types at stage 4.

As suggested, we provided the stage 4 *in situ* hybridization as Supplementary Figure 5. As can be seen, the highest expression of *Msd1* is in anthers at stage 4. At this stage, the expression level of *Msd1* in the ovaries was very low.

Because the PS and SS are separable only at stage 4 or later, we believe that organ fate determination occurs at stage 3 or earlier. Then the difference in development of PS between WT and *msd1* mutants was observed, the organ determination was already completed at stage 4 and 5. Another reason that highest expression of *Msd1* in stage 4 is because the highest expression level was observed from PS only. At stage 4, the earliest stage when it is possible to manually separate PS from SS, the average weight of PS was about 0.3 mg, whereas SS weighed about 1.7 mg. Also PS lagged slightly behind SS in development. In *Arabidopsis*, JA is critical to pollen development (Stintzi et al., *PNAS*, 2000). The *Arabidopsis* male-sterile mutant, *opr3*, lacks the 12-oxophytodienoic acid reductase required for jasmonate synthesis (McConn *et al., PNAS,* 1997). JA may play similar roles in sorghum, but this question is beyond the reach of these data. The expression of *Msd1* in anthers may have also contributed to the high expression level observed in stage 4.

2. More regarding the in situ expression data, lines 100-102: "Expression was maintained within floral organ primordia until the floral meristem initiated, and then expanded to the tip of floral meristem, including the glume, at stage 3". I thought that floral organ primordia produce floral organs; and that floral meristems are the ones that initiate flowers. So, I believe that floral meristems should precede floral organ primordia, should not they? The author's statement implies that floral organ primordia appear before floral meristem. Are they using the wrong terminology here or I am missing something?

Thank you for the correction. We changed the sentence 'Expression was maintained at the tip of the floral meristem, and then expanded throughout the floral meristem including the glume, at stage 3 (Fig. 3f–k)'

3. In general, the description of the in situ hybridization patterns could benefit of a clearer presentation/explanation.

Thanks. We added *in situ* hybridization data to supplementary Fig. 5, and provide a detailed description in the main text: "When using an antisense probe, expression signal was observed in stage 2 in WT plants, localized to the tip of the spikelet meristem (Fig. 3d–e). Expression was maintained at the tip of the floral meristem, and then expanded throughout the floral meristem

including the glume at stage 3 (Fig. 3f–h). Both transverse and longitudinal sections revealed that *Msd1* expression was specifically maintained in a specific dome-like domain of the floral meristem (Fig. 3f–k). At stage 4 (Supplementary Fig. 5), although weak expression of *Msd1* was detected in the ovary of both PS and SS, a much stronger signal was observed in anthers."

4. I do not understand how Supplemental Figure 4 supports that MSD1 is "a class II TCP transcription factor belonging to the CYC/TB1 subgroup" (lines 86-87 of the text). That figure is just a phylogeny and alignment of TCPs within sorghum. Are there any 'defining' features in the alignment that support that classification? Or is the classification supported by MSD1's homology to specific orthologs in other species? If the later, then those orthologs have to be included in the phylogeny and the defining features of the subgroup clarified in the alignment.

Suppl. Fig 4 in the previous version showed phylogenetic tree and the conserved TCP domain sequence, which was used to determine in which subgroup the *Msd1* gene belongs. As suggested by Reviewer #1, Francis et al. (2016) (https://www.nature.com/articles/srep38488.pdf) performed detailed phylogenetics analysis of the TCP family in sorghum. Therefore, we cited that paper and removed this figure.

5. Additionally, why limit the phylogenetic analysis to the grasses in Suppl. Fig. 5? Are the TCPs too different in the dicots? I think it would be more interesting and informative to know if the amino acids affected in the msd1 mutant allelic series are also conserved (and presumably important) in other orthologous TCPs beyond the grasses. In sum, I believe Suppl. Figures 4 and 5 could be made into a single figure with a proper phylogeny and alignment containing less grasses and paralogous sequences and more orthologous sequences beyond the grasses, from the dicots if possible.

As indicated in our response to the previous comment, we cited Francis et al. (2016), which reported that the *Msd1* gene (*SbTCP16*) is specific to monocots, and that *Arabidopsis* has no protein in that clade (Figure 6 from Francis et al., 2016). This is now stated in the main manuscript. The old Suppl. Fig 5b is Suppl. Fig 4 in the revised version.

6. It would also be useful to mark the location of barley INTERMEDIUM C in the phylogenetic tree in Supplemental Figure 5, to have an idea of how close to MSD1 is.

The old Suppl. Fig5a was excluded from this revised version to avoid redundancy with the Francis et al. (2016). The barley *INTERMEDIUM C* (*INT*-C) gene belongs to the same group (TCP class II, CYC/TB1 subgroup) as the *Msd1* gene, but the two genes are not from the same homology group. Barley *INT*-C gene is the ortholog of sorghum *Tb1* (*Sb01g010690*), not *Msd1*. At the amino acid level, barley INT-C has 58.72% identity with sorghum Tb1, but only 33.62% with MSD1. A new phylogenetic tree with INT-C was added as Supplementary Fig. 7.

7. Lines 121-122: I believe that at this point the authors need to define better what an 'Msd1 target gene' is. The method section entitled "Identification of putative regulatory targets of Msd1" should be part of the main text here because it is important information to understand the results presented in Figure 4.

We agree. Accordingly, we moved part of the Methods section to the Results section on Page 7.

8. Even more importantly: The pattern of expression of JA-related genes should be explained/emphasized in the main text (somewhere between lines122-127). Only in the legend to figure 4, the authors state that "Genes involved in biosynthesis were down-regulated at stage 4 in PS of msd1". This information is very important evidence (and one of the central findings of this work), so it should be explicitly stated in the text, not just in a figure legend. In lines 125-127 the authors simply state that JA biosynthesis genes follow "a pattern consistent with Msd1 regulation". However, as stated above, the explanation of what this "pattern" means was left down in the methods and it is not clear/obvious where it matters in the main text.

We agree. More details, including the pattern of the *Msd1* regulatory targets and the expression changes in JA pathway genes have been added to that section.

9. Lines 129-130. "…we measured the changes induced by JA". I believe the authors measured "the changes in JA levels", not any changes induced by JA.

That is correct. We have clarified the text accordingly.

10. Lines 134-137: "We also noted that prolonged methyl-JA treatments at concentrations greater than 1 mM decreased overall panicle size and branching compared to controls, consistent with the observation that msd1 mutants tended to have much larger panicles than WT from our previous study". With this long statement, the authors seem to imply a relationship between JA levels and panicle size, based on the observation of smaller panicles with higher-than-1mM JA treatments. However, in the method section, lines 335-3m36 the authors clearly state that higher-than-1mM JA concentrations "severely retarded growth and development of the plant". Therefore, I do not think any conclusions should/can be drawn from such high concentration treatments.

We agreed and revised the section on page 8 as suggested.

11. Lines 142-144. I think it is relevant to cite Acosta et al, 2009 (Science, 323: 262-5. PubMed ID: 19131630), which more specifically suggests a role for jasmonate in flower organ abortion also probably via programmed cell death.

Yes, this paper is now discussed on page 10.

12. Line 148. I fail to see how figure 4a supports the statement about high expression of embryogenesis-related genes in the msd1 mutant.

A: Thank you for pointing this out. The figure citation was incorrect; and we should have cited supplementary Table 4. This has been updated in the revised manuscript.

13. Supplementary Figure 8. It would be preferable to plot the qRT-PCR data in the same fashion as in Figure 4a. It is so much harder to read the data with yet a different plot display! Additionally, the plots in Suppl. Figure 8 create the false idea of developmental time in the x-axis, while in reality two different tissue types (PS and SS) are mixed together. Additionally: a) What are the samples S1 and S3 in that experiment? Only PS or only SS or a mix of both at that stage?

As shown in supplementary Fig 1, we defined the developmental stages for inflorescence architecture. In case of stages 1 and 3, we used the whole inflorescence for RNA seq and qRT-PCR. In stage 1, we could not dissect PS and SS because they had not yet distinctly developed, so we could only observe spikelet meristem. In stage 3, we could observe PS (blue asterisks) and SS (red asterisks) by SEM, but it was impossible to manually separate these tissues.

b) Can the authors provide an explanation to the differences in gene expression quantified by RNA-seq vs. qRT-PCR? For example, in Figure 4a Msd1 expression in stage 4 msd1 mutant SS is very low relative to the PS of both wild-type and mutant. However, in suppl. Fig. 8, stage 4 msd1 mutant SS displays higher Msd1 expression than mutant stage 4 PS.

We agree that the expression pattern observed by RT-PCR was not exactly consistent with the RNA-seq data. The discrepancy is partly due to the difficulty of finding a good control for both WT and mutants. To validate RNA seq, we initially collect 20 available sorghum controls (Reddy et al., Frontiers in plant science, 2016) and tested them by RT-PCR. Ultimately, we designed an experiment to test 8 different primer sets in tissue types that are commonly used for RT-PCR, and chose the best control for sorghum, *Sb-EIF4α* (Eukaryotic initiation factor 4α, *Sb04g003390*) based on its expression in various tissues of the wild type, as well as the Ct value. Expression of *Sb-EIF4α* in SS was ~1.4-fold higher in the *msd1* mutant than in the wild type, after we checked again RNA-seq data in SS. The higher expression of Sb-EIF4α at msd1 mutant SS make difference in SS at stage 4 between RNA seq and RT-PCR data.

Because of the low reliability of the RT-RCR, we decided to eliminate this supplementary Figure from the manuscript. On the other hand, we have confidence in the quality of the RNA-seq data, which were obtained in three biological replicates with an average Pearson correlation coefficient of 0.97 (Supplementary Figure 6).

14. Line 155. "… by de-repression of PS development in sorghum panicles through blockage of JA-induced programmed cell death". This is an overstatement: No evidence indicating that JA is promoting cell death is presented in this manuscript. Additionally, the involvement of cell death is only circumstantial (from the RNA-seq data); further evidence supporting a causative role of cell death in PS suppression is not presented. Therefore, the text in line 155 should probably read something like"…by de-repression of PS development in sorghum panicles through blockage of JA signaling and possibly programmed cell death".

Thank you for these comments. From the expression data, LEA genes are the most highly up-regulated group at stage 4 PS in the *msd1* mutant. Based on similar suggestions from the other two reviewers that our evidence about the PCD pathway was not very strong, we decided to eliminate the model in the original Figure 5. Instead of a figure, we describe our hypothetical regulatory model in the Discussion section on page 10.

15. It may be worth including a line or two in the discussion to cite Schommer et al, 2008 (PLoS Biol.6(9):e230. PubMed ID: 18816164). That work was the first to suggest a role for class II TCP transcription factors in promoting JA biosynthesis.

This paper has been cited in the revised manuscript on page 10.

16. Another reference that may need citation is Cai et al, 2014 (https://www.nature.com/articles/ncomms4476), which reported that JA is required for early spikelet development in rice (flower meristem determinacy and floral organ identity). This is in contrast to the negative role of JA later in floral organ development reported here by Jiao et al.

This paper has been cited in the Introduction on page 3.

17. In the legend to figures 1j-m, there is no clarification about the inflorescence stage that is displayed.

Figure 1j–m refers to stage 4. This information has been added to the legend.

Minor issues

18. In figure 1j-m, are the black lines 'surrounding' the ovary actually part of the tissue structure? Or were they added 'artificially' to emphasize the contour of the carpel? Please explain in the legend.

A clearing method was used to visualize the floral organ in SS and PS. After clearing, the carpel is quite transparent, and it is not easy to see the ovary structure inside PS and SS. To help the

reader understand the figure, we artificially added a line to emphasize the contour of the carpel in Figure 1j–m. An explanation has also been added to the legend.

19. In the ‘pipeline’ graph of figure 2a, one panel seems repeated; or is that step actually done twice in the actual pipeline?
A: Thank you for detecting this error, which arose when we were editing the figure. The appropriate correction has been made.

20. The legend to Suppl. Fig. 3 seems incorrect (panel labeling does not correspond to legend).

Thank you for pointing this out. That figure legend has been corrected.

21. The figure legend to Supplemental Figure 5 is incomplete
A: Supplement Figure 5a was eliminated, as suggested by two reviewers. The old Supplementary Figure 5b was kept as Supplementary Figure 4 with an updated legend.

22. Line 59: “...to develop…” should/can be erased.
This has been corrected in the revised version.

23. Line 114, incorrect citation, not supplementary figure 4.
Thank you. We have corrected it to Supplementary Table 5.

24. Line 116. “…the identities of the genes”. I think the authors mean “the number of the genes”.
Thank you for catching this error. The revision has been made.

<u>Reviewers' comments</u>:

Reviewer #1 (Remarks to the Author):

Much improved, thanks!

minor points and typos:
-L041, correct "… attractive functional…"
-Suppl. Fig 1, figure letters a-…, are missing in the figure
-L201, correct "…fully develops…"
-L216 ff., correct "two-rowed"
-L217 ff., correct "six-rowed"
-L229, correct "… et al. …"

Reviewer #2 (Remarks to the Author):

The authors have clarified important issues and questions raised by the reviewers, broadened and focused the discussion and added helpful citations. No additional experimental data was provided that would have clarified some of the questions raised, but even so, the discovery of the gene corresponding to MSD1 is a valuable contribution to our understanding of sorghum seed developmental biology.

The new text has several odd word uses that the authors may want to review.

l. 41 function genomics... maybe the authors intended to use the phrase functional genomics or comparative genomics?

l 63: Do the authors mean "the regulation of JA synthesis"?

l. 213: tillering?

Reviewer #3 (Remarks to the Author):

The manuscript by Jiao et al. has greatly improved after taking into account the reviewers' comments. It now looks much closer to the final form expected of a publication in Nature Communications.

However, I still believe that additional improvement is required regarding the presentation and discussion of the in situ hybridization data, as follows. If this is properly taken care of, I would have no further reservations with this manuscript.

1) I agree with the authors that the critical stage for the action of MSD1 in determining the fate of the PS (arrest) is probably stage 3. Therefore, it is indeed more important to display the in situ hybridization data at that stage and not at the stage suggested by us reviewers (stage 4).

2) However, the authors did not respond to my suggestion of clearly indicating in their in situ hybridization figure, which primordia correspond to PS and SS, particularly at stage 3 (figure 3f to 3k) since it is the most important. It is critical to assess if there is a difference in MSD1 expression between PS and SS primordia at that stage. Is MSD1 exclusively present in PS? If yes, this would nicely agree with a specific role for MSD1 in arresting PS development. On the contrary, if MSD1 expression seems the same in both PS and SS, it is important to point this out and mention that it remains an open question how MSD1 is able to specifically arrest PS development even if it is expressed in both types of spikelet. There would be nothing wrong if this is the case because the genetics clearly supports the function of MSD1.

In sum, the authors should not just limit themselves to describe the in situ hybridization patterns at stage 3. In its current form, this data is not adding anything meaningful to the manuscript. They should try to explain what these patterns may mean for the biological function of MSD1. In fact, in the discussion the authors' model of MDS1 function states that "a development signal during stage 3 activates the expression of Msd1 in PS". This hypothesis can already be tested easily with the in situ hybridization data that the authors have. Again, if the data does not support the hypothesis, then simply the model is not correct and it has to be re-written and left more open because it would be less clear how MSD1 specifically arrest PS development.

3) In lines 238-241 there is a smaller issue that needs to be re-checked. I believe that the authors are trying to argue that embryo development is occurring in SS but not in PS at stage 4. It seems to me that at that stage the reproductive organs are still quite young and anthesis will happen much, much later, am I right? Therefore, I believe that there is no such thing as seed, grain or embryo development at stage 4. If anything, perhaps there is only ovule development at that stage and even that I cannot tell for sure. Therefore, the upregulation of LEA expression in msd1 mutant probably has a functional meaning that is not directly related to embryogenesis. I suggest to modify/correct the discussion accordingly or remove altogether any mention of the LEA gene upregulation.

4) Another issue is that the new discussion text has many typos and grammatical errors.

Responses to Reviewers:

Reviewer #1 (Remarks to the

Author): Much improved, thanks!

minor points and typos:
-L041, correct "… attractive functional…"
-Suppl. Fig 1, figure letters a-…, are missing in the figure
-L201, correct "…fully develops…"
-L216 ff., correct "two-rowed"
-L217 ff., correct "six-rowed"
-L229, correct "… et al. …"

Response: Thank you! We have corrected all of them.

Reviewer #2 (Remarks to the Author):

The authors have clarified important issues and questions raised by the reviewers, broadened and focused the discussion and added helpful citations. No additional experimental data was provided that would have clarified some of the questions raised, but even so, the discovery of the gene corresponding to MSD1 is a valuable contribution to our understanding of sorghum seed developmental biology.

The new text has several odd word uses that the authors may want to review.

l. 41 function genomics... maybe the authors intended to use the phrase functional genomics or comparative genomics?
Response: Yes, we have corrected it to "functional genomics".

l 63: Do the authors mean "the regulation of JA synthesis"?
Response: Yes, the correction has been made as suggested.

l. 213: tillering?
Response: Yes, it has been changed to "vegetative tillering".

Reviewer #3 (Remarks to the Author):

The manuscript by Jiao et al. has greatly improved after taking into account the reviewers' comments. It now looks much closer to the final form expected of a publication in Nature Communications.

However, I still believe that additional improvement is required regarding the presentation and discussion of the in situ hybridization data, as follows. If this is properly taken care of, I would have no further reservations with this manuscript.
1) I agree with the authors that the critical stage for the action of MSD1 in determining the

fate of the PS (arrest) is probably stage 3. Therefore, it is indeed more important to display the in situ hybridization data at that stage and not at the stage suggested by us reviewers (stage 4).
Response: Thank you! That is also why we put the in situ of stage 4 into the supplementary file.

2) However, the authors did not respond to my suggestion of clearly indicating in their in situ hybridization figure, which primordia correspond to PS and SS, particularly at stage 3 (figure 3f to 3k) since it is the most important. It is critical to assess if there is a difference in MSD1 expression between PS and SS primordia at that stage. Is MSD1 exclusively present in PS? If yes, this would nicely agree with a specific role for MSD1 in arresting PS development. On the contrary, if MSD1 expression seems the same in both PS and SS, it is important to point this out and mention that it remains an open question how MSD1 is able to specifically arrest PS development even if it is expressed in both types of spikelet. There would be nothing wrong if this is the case because the genetics clearly supports the function of MSD1.
Response: At stage 3, the PS and SS are still not separable for in situ experiment. So we don't have the answer about whether *MSD1* exclusively present in PS or not.

In sum, the authors should not just limit themselves to describe the in situ hybridization patterns at stage 3. In its current form, this data is not adding anything meaningful to the manuscript. They should try to explain what these patterns may mean for the biological function of MSD1. In fact, in the discussion the authors' model of MDS1 function states that "a development signal during stage 3 activates the expression of Msd1 in PS". This hypothesis can already be tested easily with the in situ hybridization data that the authors have. Again, if the data does not support the hypothesis, then simply the model is not correct and it has to be re-written and left more open because it would be less clear how MSD1 specifically arrest PS development.
Response: Because we don't have further evidence about expression of *MSD1* in PS and SS at stage 3, the hypothesis has been modified more open.

3) In lines 238-241 there is a smaller issue that needs to be re-checked. I believe that the authors are trying to argue that embryo development is occurring in SS but not in PS at stage 4. It seems to me that at that stage the reproductive organs are still quite young and anthesis will happen much, much later, am I right? Therefore, I believe that there is no such thing as seed, grain or embryo development at stage 4. If anything, perhaps there is only ovule development at that stage and even that I cannot tell for sure. Therefore, the upregulation of LEA expression in msd1 mutant probably has a functional meaning that is not directly related to embryogenesis. I suggest to modify/correct the discussion accordingly or remove altogether any mention of the LEA gene upregulation.
Response: Thank you for pointing this out. We didn't make it clear in the lines 238-241 in the last version. Our hypothesis is the fates of PS and SS are determined at stage 3 and the phenotypes are developed at stage 4. In stage 4, we saw the LEA genes largely up-regulated in the *msd1* mutants in PS. This section was also modified as suggested.

4) Another issue is that the new discussion text has many typos and grammatical errors.
Response: We have carefully polished the language in the manuscript, especially the discussion part.
